# Pathways for enhancing service capability of primary healthcare institutions: a dynamic qualitative comparative analysis

**DOI:** 10.3389/fpubh.2026.1791172

**Published:** 2026-04-23

**Authors:** Meijiao Wang, Hongxiao Zhu, Shiru Duan, Yang Ma, Wanxin Cui

**Affiliations:** School of Public Administration and Humanities, Dalian Maritime University, Dalian, China

**Keywords:** configuration pathways, dynamic qualitative comparative analysis, primary healthcare institutions, service capability, spatiotemporal evolution

## Abstract

**Background:**

China has established the world's largest medical service system, with primary healthcare institutions (PHIs) undertaking over half of the country's outpatient services. However, PHIs still face multiple challenges in service capability, particularly the structural imbalance between the inverted triangular healthcare supply and the positive triangular demand. Consequently, improving the service capability of primary healthcare has become the key to advancing graded diagnosis and treatment and optimizing the allocation of medical resources.

**Objective:**

This study aims to explore pathways for enhancing the service capability of PHIs across China's provinces, identify the key conditions for achieving high-quality services, and thereby lay a foundation for policy-making.

**Method:**

Using panel data from 30 Chinese provinces covering the period 2016–2021, this study employs the TOE-DC theoretical framework, the rank-sum ratio (RSR) and the Dynamic Qualitative Comparative Analysis (QCA) method to analyze the configurational pathways and spatiotemporal evolution characteristics. Within the Technology-Organization-Environment (TOE) framework, the influencing factors are identified as technological innovation and infrastructure (technology), the healthcare workforce and financial investment (organization), and policy support, economic development, and public demands (environment).

**Result:**

(1) No single condition constitutes a necessary condition for high service capability, but the necessity of talent pools and economic growth has gradually increased over time; (2) Three pathways to achieve high service capability have been identified; (3) There is a clear spatiotemporal distribution of these pathways— the eastern region mainly adopts the government's technical coordination pathway, while the central and western regions rely more on the comprehensive configuration pathway.

**Conclusion:**

Future policies should prioritize regionally differentiated pathway selection. Each region can integrate the advantages of government macroeconomic regulation and market micro-vitality, and the government should focus on strengthening services through technical support, enhancing the monitoring of health data and disease trends, and formulating and effectively implementing forward-looking strategic plans.

## 1 Introduction

Universal Health Coverage (UHC) is the core goal of global public health governance, and its definition is to “ensure that all individuals have access to necessary health services without financial difficulties” (1). This goal has been formally endorsed by global agreements, including the World Health Assembly and the 2018 Astana Declaration, which collectively position UHC as the foundation for strengthening the global primary health care system. At the same time, these primary health care systems face the challenges of aging and chronic disease burden, which are the key drivers of morbidity and mortality (2). The global situation of achieving UHC is still challenging. As reported, approximately 4.5 billion people worldwide lacked comprehensive access to essential health services in 2021 (3). This gap is particularly serious in developing countries, where primary health care is under increasing pressure. Notably, the referral system often breaks down due to the lack of first contact ability in the primary stage. This breakdown leads to excessive concentration of hospital care, trapping scarce resources in inefficient cycles (4).

Primary health care services are the core carrier of the hierarchical medical system. The orderly operation of the hierarchical medical system relies on the improvement of the capabilities of primary health care services. However, the current Chinese hierarchical medical system is facing challenges such as weak functions of primary gatekeepers and incomplete two-way referral mechanisms. These challenges can be gradually alleviated through measures such as strengthening primary healthcare staff training, reforming medical insurance payment models, and enhancing medical information sharing (5). Research shows that the poor provision of primary healthcare services exacerbates structural inequities. For one thing, the health human resource capacity crisis characterized by general shortage and uneven distribution is the main obstacle to effective service delivery. The research reveals that the total number of medical workers in China has been steadily increasing, but there is an imbalance in regional and urban-rural distribution, and insufficient allocation at the grassroots level (6). Primary healthcare positions are typically characterized by low salaries, limited career development, and inadequate retirement benefits. These factors make it difficult to retain qualified healthcare professionals, further exacerbating the labor crisis (7). For another, outdated diagnostic equipment and limited testing tools hinder timely and effective care. This problem is particularly evident in clinics with weaker operational capabilities (8).

Faced with urgent challenges shared by developing countries, China provides an instructive case through the evolution of its primary healthcare system. As of the end of 2023, there were about 1,016,200 primary healthcare institutions (PHIs) in China. These institutions employ a workforce of 4.953 million and handle around 4.94 billion outpatient visits annually. This accounts for 51.8% of the country's total outpatient volume and brings China close to achieving universal primary healthcare coverage (9). However, compared to hospitals, PHIs still faces systemic challenges. Difficulties include a general shortage of qualified personnel (10), outdated and inadequate infrastructure (11), and limited financial resources (12). These restrictions lead patients to bypass primary facilities and go directly to large hospitals, even if their condition is very mild. This phenomenon is a mismatch between the “inverted triangle” structure of service supply and the “regular triangle” pattern of medical demand (13). Therefore, improving the capability of primary healthcare is essential to address this supply-demand gap and promote policies such as two-way referral and stratified diagnosis and treatment. China's experience in building primary healthcare provides valuable insights for other countries.

Strengthening primary healthcare capability involves non-linear and time-sensitive interactions. However, most of the existing studies focus on isolated factors, ignoring the interdependence of the system and the changes of components over time. By combining qualitative and quantitative methods, Qualitative comparative analysis (QCA) identifies asymmetric causal relationships from configurations and outcomes (14). Significantly, when used with temporal analysis or panel data, QCA further reveals that various configurations evolve as institutions develop (15). This study uses the provincial panel data of the construction and development of PHIs in 30 provinces of China (includes autonomous regions and municipalities directly under the Central Government, with the Hong Kong Special Administrative Region, Macao Special Administrative Region, Taiwan region, and Tibet Autonomous Region excluded from the research scope). In this study, dynamic QCA was used to examine the allocation conditions for improving the service capability of PHIs, which can provide a solid theoretical basis and practical guidance for various regional environments.

## 2 Literature review

The country's attention to PHIs is increasing. At the same time, academic research on the determining factors and improvement strategies of its service capability is constantly increasing. Existing research on this topic can be roughly divided into input and output categories. This study establishes a TOE-DC analysis framework based on both input and output perspectives.

### 2.1 Definition and methods of service capability of PHIs

Current research lacks a unified definition of the medical service capability of primary healthcare institutions (PHIs). Research evaluating the service capability of PHIs typically defines this capability based on institutional functional roles (16). Scholars often categorize the service capability of PHIs into medical service capability and public health service capability based on their functions and responsibilities (17, 18).

In measuring “capability,” the literature presents two primary paradigms. The World Health Organization (WHO) health systems believe that capability includes “inputs,” “processes” and “outputs,” and to relate these to indicators of “outcome” (19). The first, based on resource endowment, is known as “potential capability” (what an institution “has”) (20). It assesses the theoretical maximum service level through input indicators such as the number of medical staff, beds, total equipment value, and office space. The second, based on output performance, is termed “actualized capability (or named the result service capability)” (what an institution “does”) (17, 21), which measures output quantity (e.g., outpatient visits) and output efficiency (e.g., bed occupancy rate, physician workload).

This study chose the latter, a more dynamic perspective. Firstly, potential capability may lead to a “resource idleness” bias. That is, even if PHIs possess advanced equipment, without staff turnover or patient distrust, these resources may become “sunk costs.” Secondly, the static attributes of potential capability cannot reflect resource utilization efficiency. For example, two health centers, both with 50 beds, one operating at full capability and the other vacant year-round, would have the same “capability” under potential capability measurement.

Referring to Donabedian's Structure-Process-Outcome (S-P-O) model (22), although personnel and equipment belong to structural capabilities, they must ultimately be transformed into healthcare outputs to be considered effective capabilities. Therefore, this study selects Actualized Capability, arguing that only resources transformed into services (diagnosis and treatment, hospitalization) have social value. Furthermore, we believe that this “actualized capability” can fully reflect management effectiveness. That is, when resources are similar, institutions with higher patient volume and efficiency are considered to have better internal management, process optimization, and doctor-patient relationships. In addition, by using efficiency indicators such as bed occupancy rate and physician workload, the interference of blindly expanding quantity is eliminated, more accurately capturing the internal operational effectiveness of primary care institutions. Finally, high output means high social recognition, which is the core manifestation of service capability.

Based on the above analysis, the medical service capability in this paper is measured by medical service output, which refers to the quantity and efficiency of medical services provided by primary healthcare institutions. Inspired by Hu (23), Hu (24), and Ma (25), this study uses service output as an indicator of the service capability of PHIs.

Indicators affecting service capability include not only qualitative dimensions such as management capability, but also quantitative indicators such as service capability (26). In terms of methodology, the structural process outcome model proposed by Donabedian is still widely used in the analytical framework (27). On this basis, system tools such as the Primary Health Care Assessment Tool and QUALIOPC questionnaire can capture the core dimensions from the perspective of clinical and patients (28, 29). An important trend in these evaluations is that the concept of “patient-centered” is increasingly emphasized, and patient satisfaction and experience have become key components of the evaluation framework (30, 31). In addition, quantitative analysis methods such as TOPSIS, rank and ratio, and fuzzy set qualitative comparative analysis are also increasingly used (32–34).

### 2.2 Influencing factors and the construction of the TOE-DC framework

The TOE model, proposed by Tornatzky and Fleischer (97), provides a framework for examining the impact of technological, organizational, and environmental factors on complex governance outcomes. The service capability of PHIs is shaped by the interaction of technology, organization, and environment, rather than being driven by a single dimension. Research on the determinants of PHIs service capability can typically divide influencing factors into internal and external dimensions, a perspective that emphasizes the influence of both. The internal core elements include the healthcare workforce (10, 35), infrastructure (11), financial investment (36). The external factors include economic development (37), public demand (38), policy support (39) and technological innovation (40). Based on the TOE model, the analytical framework of this paper is as follows:

The technological dimension mainly refers to technological innovation (41, 42) and infrastructure (43, 44) in the field of primary healthcare services, such as the use of new technologies, the popularization of information technology, and telemedicine, thereby improving the accessibility and efficiency of healthcare services. Use digital tools such as telemedicine and intelligent older adult care platforms to improve service accessibility and diagnostic accuracy (41, 42). Improving infrastructure through centralized procurement and targeted investment is also key to improving the capability (45). Infrastructure development supports capability through standardized diagnostic facilities and expanded service coverage (43, 44).

The organizational dimension mainly covers the healthcare workforce (46–48) and financial investment to improve the services capability of PHIs. This involves strengthening the consistency between accessible medical education and the quality of clinical staff (49). Governments should tilt resources toward underdeveloped areas and stimulate talent development through salary reform (50).

The environmental dimension mainly includes external factors such as economic development, public demand, and policy support, describing the relevant action areas. The level of regional economic development, public health expenditure, and social conditions determine the demand for health and challenges (50). In addition, the lack of public health awareness and limited access to rehabilitation services also weaken the institutions' capability (51, 52). From an equity perspective, relevant policies should narrow regional disparities and strengthen the community service orientation of public health institutions (53).

Dynamic capability is defined as an organization's ability to integrate, build, and reconfigure internal and external competences to address rapidly changing environments (54). They emphasize that dynamic capabilities exist both within and outside an organization. This concept categorizes as a type of “capability” that is typically built rather than acquired, functioning to integrate (or coordinate), build, and reconfigure internal and external resources. These capabilities generally develop in rapidly changing environments and are heterogeneous across organizations because they depend on an organization's specific development path, unique asset base, and distinctive organizational processes. Furthermore, dynamic ability also includes the ability to sense and then seize opportunities quickly and proficiently (55). Combined with previous literature, dynamic capabilities are ultimately disaggregated into the capability (a) to sense and shape opportunities and threats, (b) to seize opportunities, and (c) to maintain competitiveness through enhancing, combining, protecting, and, when necessary (56). Some experts also emphasize that a dynamic capability is the organization's potential to systematically solve problems, formed by its propensity to sense opportunities and threats, to make timely and market-oriented decisions, and to change its resource base (57). Dynamic capability can also be used to enhance existing resource configurations in the pursuit of long-term competitive advantage (58).

**Figure 1:**
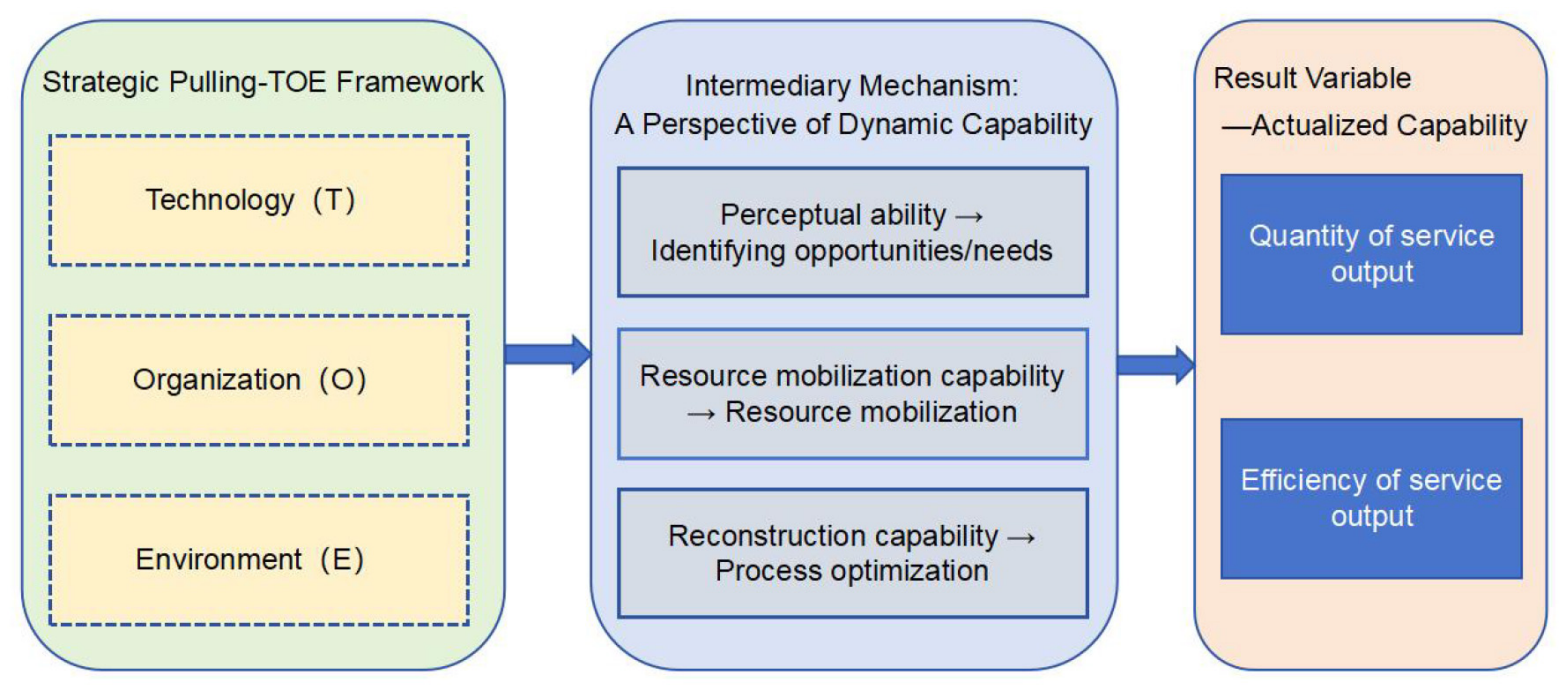
Construction of the TOE-DC framework.

As shown in Figure 1, this study constructs a framework oriented toward service capability of PHIs based on TOE-DC. This framework introduces dynamic capability (DC) as the core transformation mechanism. The organizational dimension (O) provides “potential capability” (e.g., personnel and beds); the environmental dimension (E) provides external incentives and constraints for releasing capacity (e.g., policy guidance and resident needs); and the technological dimension (T) acts as an accelerator for capacity transformation.

The dynamic capability of PHIs manifests as follows: when they perceive changes in external demand (E), they reorganize existing personnel and facilities (O) in a non-linear manner through technological means (T), ultimately achieving an increase in the number of medical services and optimization of operational efficiency at the actual capacity level. The concept of dynamic capability informs our expectation that high service capability emerges from specific, synergistic configurations of TOE conditions, which represent an institution's or region's latent capacity to sense and reconfigure resources.

Although existing studies have laid the foundation for understanding the services capability of PHIs, there are still a number of limitations. Most studies focus on analyzing the independent role of a single factor and do not pay enough attention to the complex interaction between multiple variables. In addition, existing analyses are often based on cities, and it is difficult to compare at the broader regional level. Therefore, this study employs the rank-sum ratio (RSR) method to evaluate institutional service capability and applies dynamic QCA to identify the path of high-level PHIs service capability.

## 3 Methodology

To systematically evaluate the multi-dimensional comprehensive capabilities of PHIs in 30 provinces in China, this study introduces the rank-sum ratio (RSR) method to build an evaluation framework. The RSR value will be used as the outcome variable of the QCA method. The QCA method aims to identify the conditions and configuration paths for the formation of high-level PHIs service capability.

### 3.1 Assessment of service capability in primary healthcare institutions

#### 3.1.1 RSR

In view of the complexity and abnormal value interference problems in multi-indicator analysis, this study chooses to adopt the RSR method to evaluate the comprehensive performance of regional PHIs. In the research, the entropy weight method is further introduced to construct a weighted rank sum ratio (WRSR) evaluation model. The method determines the weight according to the information entropy of each indicator itself, reducing subjective intervention, thus enhancing the reliability of the evaluation results.

#### 3.1.2 Indicator design

(1) Initial data processing and weight assignment.

The evaluation process begins with the standardization of the raw data of all indicators. For example, the number of visits to family health services in community health centers is standardized to ten thousand to ensure cross-provincial comparability. Subsequently, the entropy weight method is used to calculate the weight coefficients for each year. These coefficients reflects the dynamic contribution of each measurement indicator to the overall capability. The detailed data of variable weights are presented in Supplementary Table 1.

(2) Compilation of the rank matrix and calculation of the WRSR

This study adopts a non-integer ranking technique based on approximate linear interpolation in the ranking process. All index values are processed accordingly. This method overcomes a common limitation of the traditional RSR method. Conventional integer ranking conversion often leads to the loss of quantitative information contained in the original data. Given that all selected indicators follow a “higher-is-better” performance trajectory, ascending-order ranking was applied (59), resulting in the rank matrix R_i_, as defined in Equation 1, where n represents the number of evaluation entities. Secondly, based on the derived rank matrix and the corresponding weight coefficients of each indicator (60), the WRSR is calculated, as specified in Equation 2, where W_j_ represents the weight coefficient of the jth indicator, and R_ij_ refers to the rank of the jth indicator for the ith evaluation object. The detailed computational results are summarized in Table 1.


$$\begin{array}{ccc} R_{i} & = & 1+\left(n-1\right)\times \frac{x_{i}-x_{min}}{x_{max}-x_{min}} \end{array}$$ (1)

$$\begin{array}{ccc} {\text{WRSR}}_{i} & = & \overset{m}{\underset{j=1}{\sum }}W_{j}\frac{R_{ij}}{n} \end{array}$$ (2)

**Table 1:** Value of the WRSR by province, 2016–2021.

Province	2016	2017	2018	2019	2020	2021
Tianjin	0.260	0.242	0.238	0.234	0.185	0.191
Beijing	0.273	0.230	0.234	0.243	0.219	0.209
Hebei	0.305	0.255	0.260	0.247	0.234	0.190
Shanxi	0.194	0.172	0.159	0.156	0.164	0.148
Inner Mongolia	0.160	0.137	0.138	0.115	0.108	0.095
Liaoning	0.225	0.207	0.203	0.189	0.158	0.152
Jilin	0.110	0.109	0.116	0.113	0.113	0.118
Heilongjiang	0.164	0.152	0.119	0.122	0.089	0.093
Shanghai	0.608	0.571	0.557	0.557	0.569	0.584
Jiangsu	0.517	0.543	0.518	0.481	0.413	0.397
Zhejiang	0.436	0.424	0.435	0.435	0.375	0.426
Anhui	0.289	0.306	0.326	0.375	0.360	0.308
Fujian	0.242	0.230	0.244	0.242	0.277	0.275
Jiangxi	0.248	0.242	0.240	0.221	0.215	0.214
Shandong	0.429	0.384	0.395	0.382	0.400	0.448
Henan	0.425	0.401	0.400	0.411	0.417	0.403
Hubei	0.428	0.370	0.371	0.374	0.330	0.335
Hunan	0.321	0.306	0.320	0.333	0.359	0.359
Guangdong	0.520	0.505	0.537	0.527	0.445	0.410
Guangxi	0.298	0.265	0.279	0.286	0.289	0.290
Hainan	0.213	0.186	0.150	0.166	0.150	0.155
Chongqing	0.292	0.279	0.290	0.314	0.323	0.344
Sichuan	0.484	0.484	0.520	0.545	0.532	0.489
Guizhou	0.175	0.168	0.183	0.202	0.199	0.222
Yunnan	0.310	0.273	0.272	0.275	0.283	0.277
Shaanxi	0.200	0.197	0.205	0.202	0.172	0.150
Gansu	0.198	0.183	0.189	0.184	0.188	0.156
Qinghai	0.125	0.113	0.118	0.117	0.116	0.107
Ningxia	0.230	0.195	0.184	0.154	0.144	0.159
Xinjiang	0.250	0.236	0.214	0.225	0.204	0.168
Maximum value	0.608	0.571	0.557	0.557	0.569	0.584
Minimum value	0.110	0.109	0.116	0.113	0.089	0.093
Average value	0.301	0.283	0.284	0.284	0.271	0.267

(3) Presentation of indicator results.

From an overall perspective, the highest WRSR score across all years and provinces was 0.608, achieved in Shanghai in 2016. In contrast, the lowest score was 0.089, recorded in Heilongjiang Province in 2020. The average WRSR score across all observations was 0.294. Among the provinces, Shanghai, Sichuan, Jiangsu, Zhejiang, Henan, Shandong, and Anhui consistently attained relatively high rank-sum ratio scores throughout all the years. In contrast, Jilin, Qinghai, Inner Mongolia, Heilongjiang, Shanxi, and Ningxia consistently scored lower. This pattern suggests that economic factors alone do not play a decisive role in determining the service capability of PHIs.

Regarding regional effects, the 30 provinces were categorized based on their geographical locations. Empirical results show that there are regional differences in the service capability of each province. As illustrated in Table 2, the average WRSR scores in the eastern (0.350) and central (0.305) regions are higher than those in the western (0.237) and the northeast (0.142) regions.

**Table 2:** The WRSR scores and means for the four regions.

Region	2016	2017	2018	2019	2020	2021	Mean value
Eastern region	0.380	0.357	0.357	0.351	0.327	0.328	0.350
Central region	0.318	0.299	0.302	0.311	0.308	0.294	0.305
Western region	0.247	0.230	0.236	0.238	0.232	0.223	0.237
Northeast region	0.166	0.156	0.146	0.141	0.120	0.121	0.142

This regional disparity is related to the levels of economic and social development (21). In the eastern region, where economic growth and social infrastructure are more advanced, funding for PHIs tends to be more plentiful, allowing for a greater ability to attract medical personnel (61). As a result, the service capability of PHIs in this region is typically more developed.

The central region includes several populous provinces, such as Henan and Hunan, which produce significant medical service outputs. Additionally, due to the relatively even distribution of the population, the coverage efficiency is higher compared to the vast and sparsely populated western regions. This distribution also avoids the excessive service pressure caused by population over-concentration in large eastern cities.

Regarding the time effect, most provinces rankings and ratio scores showed a downward trend in 2020. This decline may be attributed to special events, notably the outbreak of the COVID-19 pandemic. During this time, PHIs were tasked with epidemic prevention and control efforts, including nucleic acid testing and isolation management. As a result, routine medical services were compromised, as it was challenging to balance essential healthcare needs with the sudden demands of public health crises, which further impacted service capability evaluations.

In addition, the WRSR change situation of each province in Supplementary Table 2 shows that although there were some small fluctuations during the study period, the overall ranking remained relatively stable. This time stability may be attributed to the simultaneous implementation of the national primary health care policy in all administrative units.

### 3.2 Data construction

#### 3.2.1 The dynamic QCA

Dynamic QCA can deeply explore the causal mechanism behind the evolution of service capability by introducing the time dimension. Compared with traditional cross-sectional research, this method uses panel data to track the configuration process of conditional variables. This helps to identify cumulative effects and phase transition effects, thus revealing the causal path (62).

The research method is not the annual cross-sectional fsQCA, nor is it the TQCA proposed by Caren and Panofsky (63). Instead, it is based on the Pooled QCA with time-varying necessity analysis within the time series QCA framework of Hino (64). Then the time dimension is systematically integrated into the necessity and sufficiency analysis using panel data (65). The core idea is: uniformly calibrate the data of all cases for all years to ensure cross-time comparability; and then, by decomposing the variables into between-group and within-group parts, separately examine the time effects and case effects of necessary conditions and configuration solutions.

Building on the panel QCA framework proposed by García-Castro et al. (65) this study applies tripartite temporal analytics via specialized R packages: (1) Pooled Analysis treating province-years as cases identifies core driving mechanisms that persist throughout capacity-building cycles; (2) Between-group Comparison comparing average conditions across provinces over time clarifies developmental divergence stemming from regional disparities; (3) Within-group Tracing examining changes over time within each province traces institutional critical transitions along the sequence: resource accumulation → service threshold breakthrough → institutionalized high-level equilibrium.

The key operations of between-group and within-group analysis are as follows:

Necessity Analysis: (1) Unified calibration: The 5th, 50th, and 95th percentiles of the full sample were used as fixed anchors to convert annual raw data into fuzzy set membership scores, ensuring temporal membership changes stem only from actual case condition variations; (2) Pooled Consistency: Aggregated consistency of each condition was calculated on the full sample to identify overall necessary conditions (>0.9), ignoring time and individual differences; (3) Between-group Consistency: Annual necessity consistency was computed by splitting the sample by year to test temporal changes (>0.9 for annual cross-sectional necessity); (4) Within-group Consistency: Positive deviations of annual observations from each case's multi-year average were retained to calculate intra-case consistency, testing internal dynamic relationships (>0.9 for strong dynamic necessity); (5) Multi-level coverage:The results of significant consistency were evaluated to measure the explanatory power of conditions as supplementary indicators.

Sufficiency Analysis: A truth table for the full sample was constructed with a frequency threshold of 2, original consistency of 0.9 and PRI of 0.8 for standard QCA to obtain intermediate configurational solutions. Temporal fluctuation amplitude of solution consistency quantified time effects (fluctuation > 0.2 for significant temporal heterogeneity). Case effects and cross-case robustness were tested by analyzing the annual stability of solution core conditions and the synchronicity between case membership in solutions and outcome variations.

Collectively, this approach provides comprehensive panel data processing across pooled, between-group, and within-group outcomes. The spatiotemporal compression characteristics inherent in primary healthcare capability development further necessitate the adoption of this methodology, which is manifested through policy-response lags (e.g., 3–5-year delayed effects of insurance reforms on resource allocation), non-linear accumulation of primary medical service capabilities and path-dependent institutional evolution.

#### 3.2.2 Data sources

In order to accurately track the development trajectory and provide a basis for policy formulation, the use of panel data is of key significance (66). Because of data availability, this study has built a data set covering 30 provinces in China from 2016 to 2021. There are significant differences in the selected cases in terms of economic development level, policy environment and geographical resources. This provides the necessary case heterogeneity basis for the application of QCA methods.

Data sources include: China Health Statistics Yearbook; National Data Platform from the National Bureau of Statistics; Blue Book of China's Informatization Development: Analysis and Forecast; China Regional Innovation Capability Evaluation Report.

#### 3.2.3 Variables description

(1) Result variables

As the “first line of defense” for public health, primary healthcare institutions (PHIs) are tasked with disease prevention, basic clinical services, and health management (38). In the absence of a unified definition, scholarly inquiry frequently operationalizes service capability through medical service output, reflecting the volume and efficiency of healthcare service.

This study is based on the methods of Hu (23), Hu (24), and Ma (25), using service output as an indicator of the service capability of PHIs. To capture the complexity of the output, the outcome variables are measured along two complementary dimensions (Table 3).

**Table 3:** Variables of pathways for enhancing service capability of PHIs.

Variant type	Variant dimension	Variant name	Measurement indicators	Data source
Result variable	Primary healthcare institutions service capability	Quantity of service output	Number of outpatient visits in primary healthcare institutions	China health statistics yearbook
			Number of admissions to primary healthcare institutions	China health statistics yearbook
			Number of home health visits by community health center (stations)	China health statistics yearbook
		Efficiency of service output	Bed occupancy rate in community health centers	China health statistics yearbook
			Bed occupancy rate in township hospital	China health statistics yearbook
			Average length of stay (ALOS)in community health centers	China health statistics yearbook
			Average length of stay (ALOS)in township hospitals	China health statistics yearbook
			Average daily outpatient visits per physician in community health center	China health statistics yearbook
			Average daily outpatient visits per physician in community health stations	China health statistics yearbook
			Average daily treatment procedures per physician in township hospitals	China health statistics yearbook
Conditional variable	Internal organizational factors	Infrastructure	Number of primary healthcare institutions per 10,000 population	National data
			Bed density in primary healthcare institutions per 10,000 population	National data
		The healthcare workforce	Number of primary healthcare workers per 10,000 population	National data
			Number of licensed physicians (including assistants) in primary healthcare institutions per 10,000 population	National data
			Number of registered nurses in primary healthcare institutions per 10,000 population	National data
		Financial investment	Financial investment in healthcare institutions/general fiscal budget expenditures	National data
	External environmental factors	Economic development	Gross regional product	National data
			Per capita disposable income	National data
		Public demand	Resident population	National data
			Per capita health expenditure	China health statistics yearbook
		Policy support	Volume of policy instruments targeting primary healthcare institutions	Pkulaw
		Technological innovation	Information index	Analysis and forecast of China's informationization trends
			Combined value of regional innovation capabilities	China's regional innovation capability evaluation report

A comprehensive variable composed of multiple indicators is calculated using the entropy method.

The quantitative dimension of medical service output. Three indicators measure the volume of healthcare service output in PHIs: Number of outpatient visits, Admissions, and Home health visits conducted by community health centers (and their affiliated stations). The data for these indicators are sourced from the China Health Statistics Yearbook for the years 2017–2022.

The efficiency dimension of medical service output. The efficiency of healthcare services in PHIs is measured by seven indicators: Bed occupancy rate in community health centers, Bed occupancy rate in township hospital, Average length of stay (ALOS) in community health centers, ALOS in township hospitals, Average daily outpatient visits per physician in community health center, Average daily outpatient visits per physician in community health stations, and Average daily treatment procedures per physician in township hospitals.

In the end, these indicators are combined into a comprehensive assessment value to represent the healthcare service capability. The data of indicators come from the Chinese Health Statistics Yearbook from 2017 to 2022.

(2) Conditional variables

The service capability of PHIs is influenced by internal organization and external environmental factors. The internal organization of PHIs is composed of organizational factors (O) with three core components: the healthcare workforce (35), infrastructure (11) and financial investment (36). These elements define the upper limit of service delivery capability. The level of infrastructure affects the feasibility of diagnosis and treatment plans (67). Financial resources can affect the stability of the pharmaceutical supply chain and the adequacy of labor (68). In addition, the size and capacity of medical personnel determine the efficiency and quality of clinical services. Fully allocating and utilizing these factors is crucial for ensuring the operation of the institution (69).

External environmental factors include environment (E) and technological (T) factors with economic development (37), public demand (38), policy support (39), and technological innovation (40). Economic development provides basic resource support. It not only determines the scale of GDP-based fiscal investment, but also promotes the participation of private capital through the market. Public demand presents dual attributes, including quantitative expansion and quality upgrading. Expectations for service quality have changed from reachability to accuracy. At the same time, the prominent pressure of demand, such as the aging of the population and the increased burden of chronic diseases, requires comprehensive improvement of services. Policy support reorganizes resource allocation through “hard constraints” and “soft incentives.” Technological innovation reconfigures the service supply chain by generating substitution and enhancement effects, thereby improving service quality and efficiency.

These antecedent variables are not randomly selected. Among them, organizational factors (O) provide the fundamental support, the external environment (E) offers pressure and guidance, and technological factors (T) act as catalysts. Together, they form a complex driving system for primary healthcare institutions to enhance their service output.

In QCA analysis, DC is not modeled as a separate, measurable condition but rather as an interpretive lens for the configurations. At this time the TOE-DC framework is a theoretical heuristic used to select and categorize the seven antecedent conditions and to interpret the resulting configurations, not a model where DC is an additional variable in the truth table.

(3) Variable preprocessing and calibration

First, the data units are standardized to mitigate the impact of regional population differences on the output of medical services. This was achieved by normalizing key indicators—including the number of PHIs, the number of beds, healthcare professionals, practicing (assistant) physicians, and registered nurses—into per 10,000 population metrics. Secondly, the entropy value method was applied to comprehensively assess infrastructure, talent development, economic growth, public healthcare demand, and technological innovation. The comprehensive measurement results are shown in Supplementary Table 3.

Furthermore, this study applied the direct calibration method to standardize all variables, with the 95th percentile, 50th percentile, and 5th percentile serving as calibration anchors, which correspond to full membership, the cross-over point, and full non-membership, respectively (70). The calibration thresholds for each variable are summarized in Table 4. The calibration results are shown in Supplementary Table 4.

**Table 4:** Calibration of variables.

Variable name			Calibration		
			Full membership	The crossover point	Full non-membership
Outcome variable	Y	PHIs service capability	0.532	0.243	0.113
Conditional variable	X1	Infrastructure	0.792	0.574	0.145
	X2	The healthcare workforce	0.678	0.392	0.162
	X3	Financial investment	0.103	0.086	0.057
	X4	Economic development	0.723	0.239	0.087
	X5	Public demand	0.605	0.297	0.105
	X6	Policy support	0.501	0.117	0.016
	X7	Technological innovation	0.600	0.135	0.046

This study used the 5th, 50th, and 95th percentiles as calibration anchors. This is based on data distribution characteristics, aiming to cover the complete range of conditional membership degrees while ensuring that low, medium, and high cases are fully represented. When there is a lack of established external theoretical standards, percentile based calibration is a common practice in QCA methods (71–73). Among them, the 50th percentile (median) serves as the intersection point, which has statistical natural significance. The 5th and 95th percentiles correspond to cases with extremely low and high membership degrees, respectively, which helps to avoid the interference of extreme outliers.

## 4 Data analysis and empirical results

### 4.1 Necessity analysis of single condition

Prior to conducting sufficiency analysis, we examined whether all individual conditions —or its absence— serve as necessary conditions for high or low healthcare service capability. In line with QCA principles, a condition can be identified as a necessary condition for the outcome if its consistency score surpasses the threshold of 0.9 (72). Table 5 presents the raw consistency and raw coverage scores for all conditions and their negations. As displayed in Table 5, the consistency scores of all conditions are below the 0.9 threshold. Specifically, ~X7 records the highest consistency score of 0.844 for the low-capability outcome, followed by X5 with a consistency score of 0.836 for the high-capability outcome and ~X5 with 0.832 for the low-capability outcome. Although these values approach the threshold, none exceed 0.9. The consistency scores of the other conditions and their negations range from 0.45 to 0.76, all far below the cutoff value. Accordingly, no individual condition (or its negation) constitutes a necessary condition for either high or low primary healthcare service capability. This result confirms that service capability of PHIs is not determined by a single factor, but is generated by the synergistic effects of multiple conditions. It also provides a reasonable justification for adopting the QCA approach to investigate conditional configurations in the subsequent analysis.

**Table 5:** Analysis of the necessary conditions.

Conditional variable	Y				~Y			
	Raw consistency	Raw coverage	Inter-group consistency adjustment distance	Intra-group consistency adjustment distance	Raw consistency	Raw coverage	Inter-group consistency adjustment distance	Intra-group consistency adjustment distance
X1	0.606	0.609	0.076	0.487	0.665	0.700	0.027	0.404
~X1	0.701	0.667	0.067	0.404	0.628	0.625	0.092	0.454
X2	0.669	0.677	0.419	0.316	0.575	0.609	0.425	0.404
~X2	0.613	0.580	0.440	0.382	0.695	0.687	0.360	0.288
X3	0.703	0.682	0.144	0.377	0.591	0.600	0.110	0.460
~X3	0.588	0.579	0.214	0.449	0.687	0.707	0.174	0.371
X4	0.697	0.739	0.257	0.332	0.528	0.586	0.324	0.465
~X4	0.610	0.552	0.205	0.432	0.765	0.725	0.202	0.282
X5	0.836	0.826	0.119	0.277	0.499	0.516	0.141	0.521
~X5	0.510	0.493	0.153	0.526	0.832	0.842	0.113	0.288
X6	0.637	0.684	0.235	0.354	0.536	0.603	0.318	0.432
~X6	0.630	0.565	0.229	0.382	0.719	0.674	0.186	0.288
X7	0.743	0.820	0.098	0.354	0.457	0.528	0.147	0.598
~X7	0.573	0.502	0.082	0.471	0.844	0.775	0.076	0.255

An analysis was conducted on the consistency adjustment distance. The study found that both inter-group and intra-group comparison values exceed 0.2. This finding necessitates further investigation into the potential presence of significant temporal effects or regional effects (65).

The distances adjusted for inter-group consistency were greater than 0.2 in all conditions, indicating regional and case-specific effects between the condition variables and the outcome variable, resulting from the considerable disparities among the 30 sampled regions in terms of economic development, policy support, public demand, as well as the infrastructure and workforce capability of primary healthcare systems (74).

This study presents a detailed analysis of the inter-group consistency adjustment distances across all years. Several causal combinations exhibit large inter-group consistency distances, suggesting potential temporal effects. These findings are summarized in Table 6, revealing the following key results: In the analyzed situations (Situations 2, 3, 4, 5, 6, 7, 8, 10, and 12), the intergroup consistency values for all observation years were below the 0.9 threshold. Therefore, none of these situations can be regarded as a necessary condition to explain the high or low service capability of PHIs.

**Table 6:** Data between groups with adjusted distance greater than 0.2.

Situation	Causal combination situations	Intergroup metrics	Year					
			2016	2017	2018	2019	2020	2021
Situation 1	X2/Y	Intergroup consistency	0.34	0.465	0.619	0.79	0.909	0.971
		Intergroup coverage	0.899	0.805	0.772	0.705	0.607	0.563
Situation 2	~X2/Y	Intergroup consistency	0.866	0.805	0.704	0.557	0.387	0.286
		Intergroup coverage	0.593	0.556	0.579	0.616	0.579	0.549
Situation 3	~X3/Y	Intergroup consistency	0.65	0.622	0.642	0.693	0.451	0.441
		Intergroup coverage	0.641	0.593	0.583	0.586	0.518	0.519
Situation 4	X4/Y	Intergroup consistency	0.462	0.592	0.691	0.771	0.817	0.901
		Intergroup coverage	0.89	0.831	0.799	0.747	0.683	0.625
Situation 5	~X4/Y	Intergroup consistency	0.747	0.7	0.63	0.557	0.535	0.455
		Intergroup coverage	0.566	0.533	0.543	0.561	0.551	0.566
Situation 6	X6/Y	Intergroup consistency	0.806	0.787	0.536	0.585	0.569	0.505
		Intergroup coverage	0.68	0.666	0.737	0.745	0.606	0.696
Situation 7	~X6/Y	Intergroup consistency	0.469	0.474	0.719	0.695	0.724	0.732
		Intergroup coverage	0.717	0.561	0.554	0.561	0.59	0.481
Situation 8	X2/~Y	Intergroup consistency	0.292	0.373	0.494	0.661	0.758	0.811
		Intergroup coverage	0.647	0.663	0.631	0.604	0.591	0.586
Situation 9	~X2/~Y	Intergroup consistency	0.955	0.89	0.821	0.678	0.496	0.395
		Intergroup coverage	0.548	0.631	0.689	0.767	0.864	0.944
Situation 10	X4/~Y	Intergroup consistency	0.318	0.402	0.483	0.574	0.626	0.72
		Intergroup coverage	0.513	0.579	0.572	0.57	0.611	0.622
Situation 11	~X4/~Y	Intergroup consistency	0.932	0.882	0.831	0.745	0.675	0.565
		Intergroup coverage	0.592	0.69	0.734	0.769	0.811	0.876
Situation 12	X6/~Y	Intergroup consistency	0.779	0.638	0.435	0.469	0.568	0.367
		Intergroup coverage	0.552	0.555	0.613	0.612	0.706	0.63

**Figure 2:**
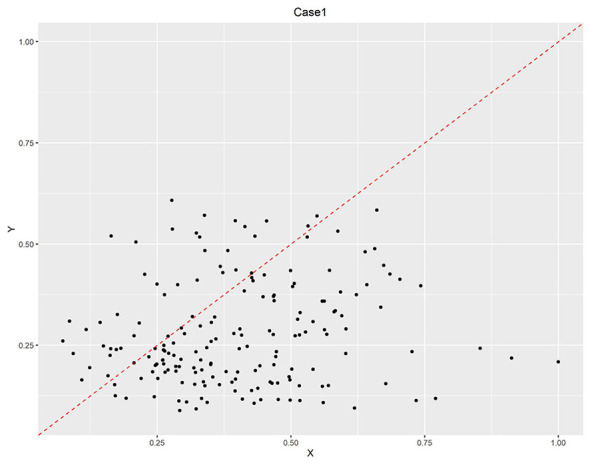
Scatter plot of necessary condition test results of case 1.

**Figure 3:**
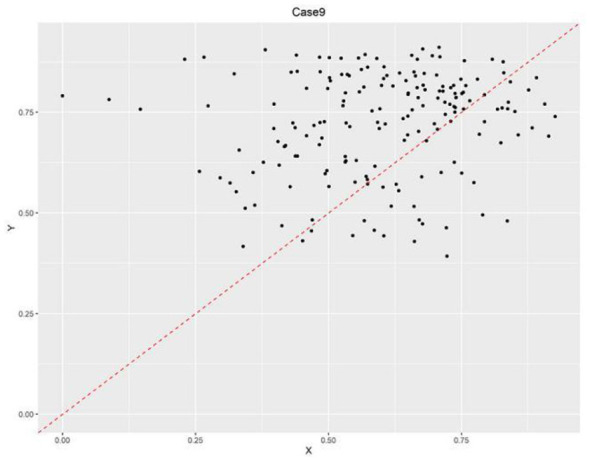
Scatter plot of necessary condition test results of case 9.

**Figure 4:**
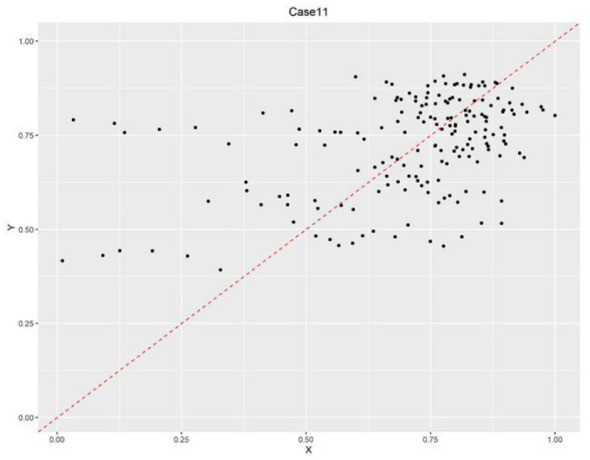
Scatter plot of necessary condition test results of case 11.

In contrast, Situations 1, 9, and 11 showed intergroup consistency coefficients ≥ 0.9 and coverage rates ≥0.5 in some years, meeting the predefined thresholds. According to the method outlined in reference (75), the X-Y scatter plots of relevant years were constructed. As illustrated in Figures 2–4, situations 9 and 11 do not meet the necessary conditions (76), as about one-third of the case points are above the diagonal. However, Situation 1 shows that a strong healthcare workforce was necessary to achieve high service capability between 2020 and 2021. This increase in necessity is likely attributable to the critical role of aligning with the social context that PHIs played following the outbreak of COVID-19 in 2020. PHIs, as front-line entities, provide epidemic prevention and control measures, including fever screening, quarantine management and vaccination. The surge in demand for primary healthcare workers accelerated talent allocation. This led to a significant increase in healthcare personnel during this period.

**Figure 5:**
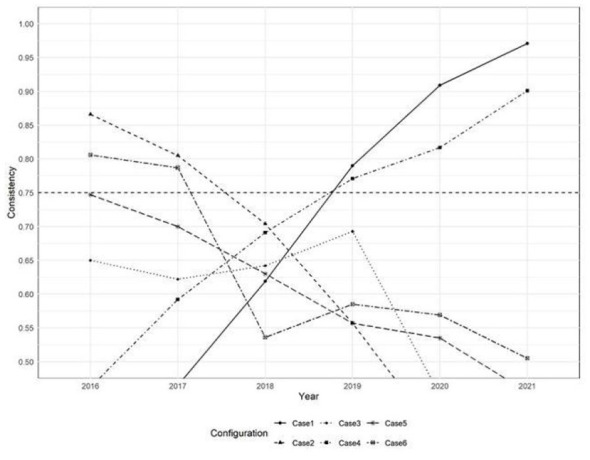
Intergroup consistency changes in case 1–6.

**Figure 6:**
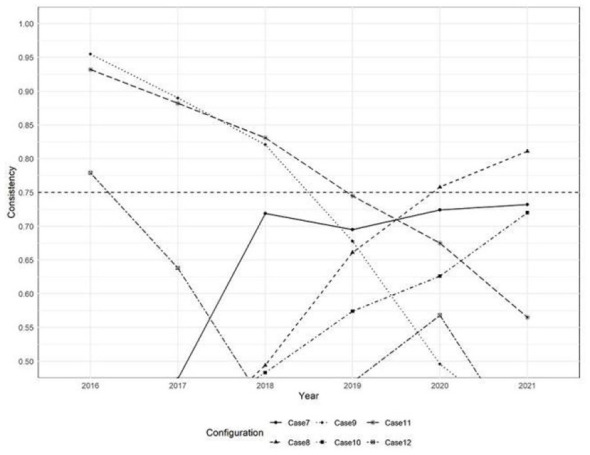
Intergroup consistency changes in case 7–12.

Regarding the temporal trends across scenarios, the changes in intergroup consistency of Situations 1–12 were visualized over time in Figures 5, 6. It is worth noting that the consistency of high personnel levels (X2) and high economic development (X4) show a clear upward trend. This indicates that the necessity of these conditions is increasing throughout the entire study period. On the contrary, the consistency of low personnel levels (~X2), low economic development (~X4), and high policy support (X6) showed a downward trajectory. Together, these temporal analyses imply that regions may improve the service capability of PHIs by prioritizing human resource development and economic growth. It should be noted that while the talent pool and economic growth do not yet constitute universally necessary conditions for the outcome, their necessity exhibits a significant and strengthening temporal effect.

### 4.2 Adequacy analysis of conditional configuration

The adequacy analysis of conditional groupings aims to evaluate how different configurations of conditions influence the outcomes. In this study, a truth table was constructed, and thresholds for consistency, case frequency, and PRI were established (72). Drawing upon prior research and based on the characteristics of the case data (77, 78), the consistency threshold for condition configurations was set at 0.9, the frequency threshold at 2, and the Proportional Reduction in Inconsistency (PRI) threshold at 0.8.

The selection of these thresholds is grounded in established methodological guidelines for QCA and the specific characteristics of the study's sample. For the frequency threshold, a value of 2 was adopted given the sample size of approximately 180 cases. This conforms to norms for large-sample QCA research, which retain only configurations backed by sufficient empirical evidence—thereby reducing the impact of outliers and enhancing the robustness of the findings. For the raw consistency threshold, a cutoff of 0.90 was applied, which exceeds the conventional minimum of 0.80 for establishing sufficient conditions (14, 79). This conservative standard ensures that the identified configurations form highly consistent subsets of the outcome. For the PRI consistency threshold, a value of 0.80 was used to resolve the problem of simultaneous subset relations (i.e., a configuration being a subset of both the outcome and its negation). This threshold is stricter than the widely recommended benchmark of 0.70 (80), guaranteeing unambiguous model solutions.

There are differences in regional conditions and resources among different provinces in China. It is challenging to determine the exact impact of antecedents on outcome variables. Therefore, no directional assumptions were applied to any conditional variables and the final dataset contains 143 cases. R language software was used to analyze the sufficiency of the condition configuration, resulting in three types of solutions: complex, simple, and intermediate. In this paper, core conditions were identified by comparing the intermediate and parsimonious solutions, while conditions that appeared exclusively in the intermediate solutions were classified as edge conditions.

Aligned with the central theme of this paper, the following section focuses on an in-depth analysis of the theoretical foundations and key characteristics of high-level configuration conditions that influence the service capability of PHIs. The results of the analysis of the conditional configuration are presented in Table 7, which identifies four distinct pathways with an pooled consistency of 0.935 (>0.8), a PRI of 0.862 (>0.7), and an pooled coverage of 0.503 (>0.5). This study identifies four solution pathways demonstrated high consistency contribute to high PHIs service capability, accounting for 26.3%, 31.7%, 25.7%, and 25.4% of cases, respectively. Collectively, they achieve significant explanatory coverage and constitute sufficient conditions for attaining high-level service capability.

**Table 7:** Results of the configuration analysis.

Condition variable	High-level service capability of primary healthcare institutions			
	Configuration 1	Configuration 2	Configuration 3	Configuration 4
Infrastructure	⊗	⊗	•	
Healthcare workforce	⊗		•	⊗
Financial investment	•	⊗	•	•
Economic development	•	•		⊗
Public demand	•	•	•	•
Policy support		•	•	•
Technological innovation	•	•	•	•
Consistency	0.959	0.926	0.969	0.969
PRI	0.859	0.829	0.895	0.881
Raw coverage	0.263	0.317	0.257	0.254
Unique coverage	0.057	0.123	0.038	0.026
Inter-group consistency adjusted distance	0.040	0.089	0.031	0.031
Intra-group consistency adjusted distance	0.144	0.161	0.132	0.115
Overall consistency	0.935			
Overall PRI	0.862			
Overall coverage	0.503			

• and ⊗ indicate the presence and absence of core conditions, respectively; • and ⊗ represent the presence and absence of edge conditions, respectively; a blank cell signifies that the condition is either present or absent.

(1) Model 1: The Demand-Technology Compensatory Pathway (Configurations 1 & 4)

H1 1 and H4 share the same core conditions—a low-level healthcare workforce, high public demand, and high technological innovation—with high financial investment as a common marginal condition. The cases are distributed across provinces including Anhui, Guangdong, Sichuan, Hunan, and Shandong, with large populations and high healthcare demand. Facing rising demand from chronic disease management and an aging population, PHIs in these provinces are strained to meet service needs.

H1 and H4 collectively present a “demand-technology compensation” model. From the TOE-DC theoretical framework, the core characteristics of these two paths can be summarized as follows: under the condition of a disadvantaged healthcare workforce (organizational factor of O), a dynamic compensation mechanism for the shortage of this core production factor is formed through the deep coupling of environmental pressure (E) and technological empowerment (T).

H1 and H4 differ structurally in their combination of conditions: The core condition of the H1 path (capital-technology compensation) is a lack of healthcare workforce, supplemented by marginal conditions of high financial investment, high economic development, high public demand, and high technological innovation. Generally, under organizational disadvantages with significant talent shortages, the path relies on a strong regional economic foundation and robust fiscal capacity to introduce advanced technology through market mechanisms. The core conditions of the H4 path (policy-technology compensation) are a lack of healthcare workforce and a low level of economic development, supplemented by marginal conditions of high financial investment, high public demand, high policy support, and technological innovation. Its characteristic is that, under the dual constraints of talent shortages and economic disadvantages, it cannot rely on market forces for technology investment, but instead relies on institutional advantages to introduce appropriate technologies through administrative means.

From the TOE-DC theoretical framework, the internal mechanism of Model 1 can be deconstructed as follows:

First, the coupling of organizational disadvantages and environmental pressures is the logical starting point for compensation. Both paths begin with the healthcare workforce shortage (organizational factor disadvantage) and are driven by high public demand (environmental factor pressure). From a dynamic capability perspective, this is an adaptive response by organizations after perceiving an imbalance between internal and external factors—the healthcare workforce shortages constitute a constraint on the supply side, while public demand constitutes a pull on the demand side.

Secondly, the functional substitution of organizational elements by technological elements is a compensatory path. In the TOE framework, technological innovation plays a crucial role in this functional substitution. Both paths compensate for healthcare workforce shortages through technological means. For example, Anhui Province hired a provincial “Intelligent Medical Assistant” to provide work support for general practitioners through automated medical record review, diagnostic assistance, and drug use guidance. Simultaneously, Guangdong Province established a telemedicine network connecting more than 4,600 institutions to provide clinical guidance and medical education to strengthen the capability of primary healthcare. This substitution mechanism can be understood from two dimensions: First, knowledge substitution, where technologies such as AI-assisted diagnosis, clinical decision support systems, digital training systems, and standardized treatment pathways accelerate the capability building of primary healthcare workers, enabling rapid accumulation of human capital; second, spatial substitution, where technologies such as telemedicine platforms break down the physical boundaries of service supply, allowing professionals to serve a wider population. From a dynamic capability perspective, this is a process of reconstructing the service supply model through the introduction of technology.

Finally, the differentiation of compensation paths stems from differentiated resource acquisition mechanisms. The H1 path (capital-technology compensation type) presents an endogenous resource acquisition mechanism. In regions with higher levels of economic development, organizations leverage their economic advantages to directly purchase advanced technologies from the market, following the logic of “market-driven, capital for technology.” The H4 path (policy-technology compensation type) presents an exogenous resource acquisition mechanism. In regions with lower levels of economic development, market forces are insufficient to support technology investment; organizations acquire technological resources through policy tools, and the compensation path follows the logic of “government-led, policy for technology.” Both paths jointly confirm the core insight of equivalence in configuration analysis—the same capability objective can be achieved through differentiated combinations of factors and resource acquisition paths.

Both paths demonstrate an organization's strategic adaptability under resource constraints: when internal core elements (the healthcare workforce) are scarce, organizations can sense environmental pressures, capture external resources (capital or policy), and restructure service models to dynamically compensate for shortcomings in production factors. This mechanism provides regions with different resource endowments with a “different paths leading to the same goal” capacity-building strategy—developed regions can rely on market forces to achieve capital-technology compensation, while underdeveloped regions can leverage institutional arrangements to achieve policy-technology compensation.

Some other studies have also confirmed this mechanism, arguing that the integration of information technology (IT) and AI has enhanced the efficiency and services capacity of PHIs (81). Telemedicine has demonstrated immediate improvements in healthcare access in rural and marginalized populations (82). In rural China, mobile health systems have been effective in managing chronic diseases, particularly in underserved populations (83), which is highly consistent with the conclusions of this study. To mitigate long-term talent shortages, leadership development and integrated care models must be underpinned by systemic policy reforms, such as financial incentives and career development paths (84–87). Especially in economically underdeveloped remote or rural areas, evidence suggests that offering career development opportunities and community recognition can significantly improve healthcare workers' retention rates (88). Furthermore, the role of the mechanism can be further strengthened by leveraging technology to empower human capital, such as enhancing healthcare access in rural and underserved areas by training local workers to use basic telemedicine technologies and mobile devices with low-bandwidth networks for video consultations and data sharing (89).

(2) Model 2: The Government-Technology Synergy Model (Configuration 2)

H2 reveals a service capacity leapfrog path centered on collaborative upgrading. With relatively well-developed traditional infrastructure, high policy support from environmental factors (E) and high technological innovation from technological factors (T) serve as core driving conditions, supplemented by marginal conditions such as high economic development and high public demand, jointly promoting a leapfrog improvement in service capabilities. Cases conforming to this configuration exhibit spatial clustering characteristics, mainly concentrated in the economically developed eastern coastal regions, including Jiangsu, Zhejiang, Shanghai, Beijing, and Tianjin.

From the TOE framework, this path addresses the challenges of digital transformation and capacity upgrading. Technologically, it leverages regional digital economic advantages to achieve deep application of medical technologies. Organizationally, it reduces incremental investment, eliminating redundant construction and allowing fiscal resources to be redirected toward purchasing services and incentivizing innovation. Environmentally, it forms a strong support model of “policy setting the direction, market supplying technology, and demand driving application.”

The core mechanism of the H2 pathway is the “dual-engine” effect of policy support and technological innovation, rather than a simple superposition. In terms of institutional incentives, the government guides technology application through regulation. In terms of technology empowerment, the market translates policy objectives into concrete effectiveness. For example, Zhejiang uses digital economy to promote wearable devices for chronic disease monitoring; Taizhou uses the UAV drug delivery system to deliver drugs to rural clinics within 10 min; Beijing has established regional prescription review centers and intelligent pharmacy systems to significantly reduce medication errors through pre-dispensing review and centralized medication allocation. From a dynamic capability perspective, this represents an organization's ability to accurately capture institutional and technological opportunities. Through effective resource allocation, it then optimizes and upgrades its service delivery model.

Research shows that a variety of technological advances hold promise for increasing capacity and quality in primary care, such as body sensors and monitors for managing health conditions (90), medication management technology including assessing indications, prescribing, dispensing, organizing, reminding, and monitoring effectiveness (91), and telehealth that bringing expertise to the patient at the point of care and overcoming spatial and temporal barriers (92). This is consistent with our research, and we further believe that the empowerment and efficiency-enhancing mechanism is achieved through three forms. First, process optimization: digital platforms restructure service processes and improve facility operational efficiency. Second, spatial extension: technologies such as telemedicine and drones overcome physical limitations and expand service boundaries. Third, resource integration: regional information platforms integrate scattered existing resources into a collaborative network.

The H2 path validates the possibility of a level leap. From the perspective of TOE-DC theory, organizations in this process develop a dynamic capability of “policy-driven and technology-enabled,” specifically manifested in cross-sectoral resource integration, service upgrade capabilities, and environmental adaptability. As confirmed by WHO, digital health supports primary health care by improving the ability to gather, analyze, manage and exchange data and information in all areas of health (93). In addition, to make digital health a reality in primary health care, the following key components are also required: building the physical infrastructure; deploying appropriate services and applications; ensuring a sound legal and regulatory environment; and improving governance, policy, standardization and interoperability (94). It can be seen that the combined effect of technology and policy is key to this path. This path establishes a positive cycle: policy support provides institutional guarantees for technological innovation, technological innovation activates the potential of existing infrastructure, and regional economic advantages and high-level demands provide a continuous driving force for the cycle.

(3) Model 3: The Comprehensive Internal-External Synergy Model (Configuration 3)

The core characteristics of configuration H3 are sound infrastructure (technological element T), a robust healthcare workforce (organizational element O), sufficient financial investment (organizational element O), strong public demand (environmental element E), strong policy support (environmental element E), and active technological innovation (technological element T). This combination presents a structural feature of balanced development and comprehensive synergy among the three elements of technology, organization, and environment.

From the TOE-DC theoretical framework, the internal mechanism of Model 3 can be deconstructed as follows:

First, the core characteristic of the H3 path is that all three elements—technology, organization, and environment—are at a high level, and they are deeply coupled rather than simply superimposed. In terms of technology, well-developed infrastructure constitutes the material carrier of technological capability, providing the underlying support for service innovation to perceive changes in external demand. Infrastructure is not only the physical condition for service provision, but also the technological foundation for organizations to perceive environmental changes and respond to public needs. From an organizational perspective, a sufficient healthcare workforce and substantial financial investment form a complex of organizational capability and strategic capability. Human capital determines the upper limit of an organization's ability to absorb, integrate, and apply technological innovation; financial investment provides continuous resource guarantees for system operation and capability iteration; together, they constitute the institutional foundation for service supply. From an environmental perspective, strong public demand provides continuous market signals to guide services; proactive policy support reduces transaction costs through institutional incentives; and cutting-edge technological innovation accelerates resource transformation through efficiency empowerment.

Second, the dynamic capability cycle drives progress. Firstly, public demand, as a continuous environmental signal, is precisely perceived by the organization's well-developed infrastructure (technological capabilities) through market selection mechanisms, forming a dynamic response that matches service supply with demand. Secondly, the perceived demand information is analyzed and interpreted by the healthcare workforce (organizational capabilities), transforming it into a basis for resource allocation decisions; proactive policy support, as an institutional environment, guides the organization to capture external opportunities through incentives and regulations; cutting-edge technological innovation provides the tools and means to capture these opportunities. Thirdly, based on decision-making, the organization integrates resources through high-intensity financial investment (strategic capabilities), and reconstructs service processes and supply models with the help of cutting-edge technological innovation (technological environment), achieving iterative upgrades in capabilities.

Third, the deeper characteristic of the H3 path is the formation of a self-reinforcing evolutionary loop. This self-mechanism can be understood from three dimensions: First, positive feedback from the demand side. Improved service capabilities lead to higher public satisfaction, enhancing public trust and compliance with primary healthcare, and further expanding the social foundation for service demand and policy support. Second, capacity accumulation on the supply side. Continuous the healthcare workforce cultivation, infrastructure upgrades, and technology application enable organizational capabilities to accumulate. The stronger the capabilities, the stronger the ability to absorb new resources and technologies. Third, continuous empowerment from the institutional side. High-level service capabilities can attract more policy attention and resource allocation, forming a virtuous cycle of “the stronger the capabilities, the more support,” further consolidating and strengthening the system's synergistic effect.

However, this comprehensive development model places extremely high demands on resource endowment and governance capabilities, resulting in a relatively limited number of cases, mainly concentrated in regions with a relatively good development foundation.

### 4.3 The spatiotemporal two-dimensional evolutionary trajectory of service capability in high-level primary healthcare institutions

**Figure 7:**
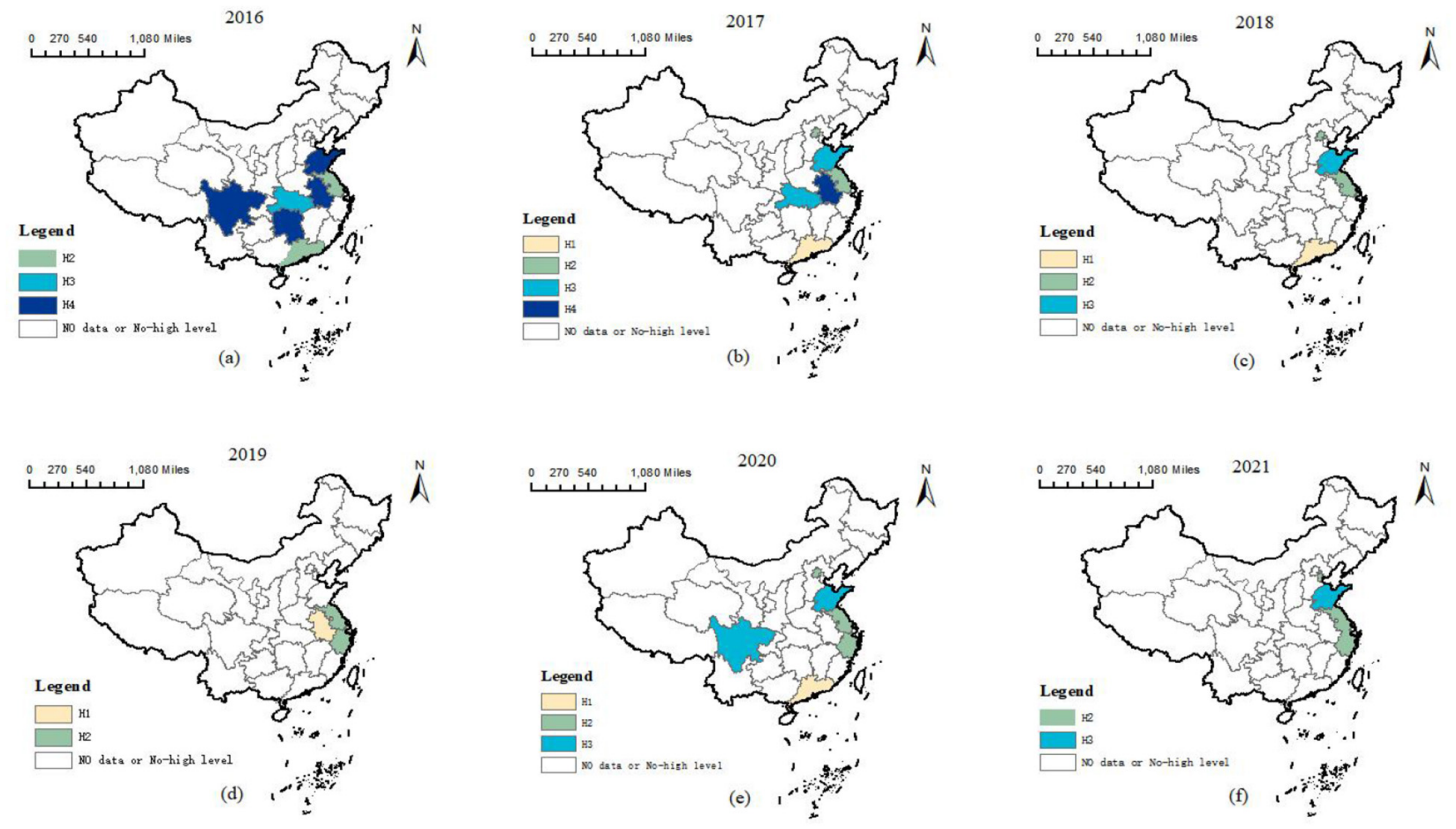
Evolutionary trajectories of service capability in high-level primary healthcare institutions (2016–2021). Subplots **(a–f)** correspond to the provinces that achieved high medical service capability coverage in the respective years 2016 through 2021.

Taking the between and within analysis results as the basis, this section explores the spatiotemporal two-dimensional evolutionary trajectory of service capability in high-level primary healthcare institutions, so as to reveal the temporal evolution and spatial differentiation characteristics of service capability. The development trajectory of the service capability of PHIs in China varies according to provinces and stages. Figure 7 shows the progress of provinces along the pathways of high-level PHIs service capability (H1, H2, H3, and H4) from 2016 to 2021. By drawing case studies representing different time pathways, the temporal and spatial effects in these pathways become more obvious. Different colors in the Figure 7 indicate four configurations, and white indicates missing data or non-high-level paths for analysis.

#### 4.3.1 Time effect analysis of service capability in high-level primary healthcare institutions

**Figure 8:**
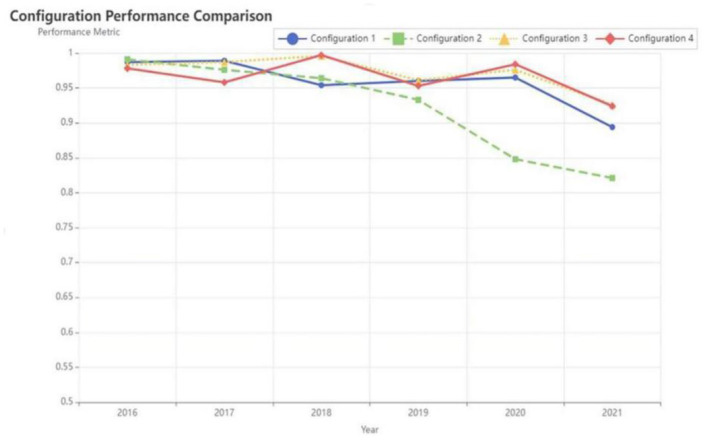
Changes in consistency levels among configurations (2016–2021).

To further examine the time effect of the service capability in PHIs, this study analyzed the inter-group consistency adjustment distance for each pathway. As shown in Table 7, the inter-group consistency adjustment distance of the four condition configurations are lower than 0.2, indicating that there was no significant time or case effect. Regarding the changes in consistency levels across different configurations in Figure 8, the consistency of each configuration fluctuates around 0.9, and a notable decrease occurred in 2020, below the threshold of 0.85. According to the evaluation results of the service capability in PHIs, this decline can be attributed to the pathway interruption caused by the mutation of the external environment. Due to unexpected events, such as the pandemic, the focus of PHIs shifted from primary healthcare services to public health emergency response in 2020. The urgent reallocation of resources to pandemic prevention and control led to reduced synergy and consistency.

Specifically, pathway H1, which was not observed in 2016, has been present and stable since 2017 and is predominantly associated with Guangdong Province. By 2019, it had also become the main way for Anhui province to develop its service capability. From 2016 to 2020, the overall consistency level of H1 remained stable. This indicates that the demand for public services and technological innovation are the initial driving forces. However, after 2020, this consistency has decreased. This means that the demand technology dual drive model may not be able to sustain long-term development. With the improvement of public demand and technical level and decline of the marginal benefit in high demand and advanced technology, the talent shortage has gradually become a limiting factor.

Since 2016, pathway H2 has been the main development route of Jiangsu province. By 2017, its influence had expanded to Beijing, and by 2019 it had further extended to the Jiangsu, Zhejiang, and Shanghai region, where it remained a stable development pathway through 2021. Although the consistency of H2 remained above the threshold of 0.75 from 2016 to 2021, it showed a gradual downward trend in general. The main root cause is that the constraint effect of “low infrastructure” and “low fiscal investment” is gradually intensifying. In the initial stage, policy and technical inputs effectively improved the capability of primary healthcare services. However, over time, the outdated infrastructure became increasingly difficult to support evolving technology applications. Therefore, the overall consistency for Configuration 2 fluctuated significantly in 2020.

Path H3 became the main route in Hubei Province in 2016 and 2017. Later, H3 evolved to represent the development trajectory of Shandong Province. From 2016 to 2018, the consistency of H3 configuration has steadily improved. This trend shows that during this period, there has been a synergistic effect between high fiscal investment, strong policy support, high public demand and advanced technological innovation, that is, the consistency of internal and external factors has been optimized. However, from 2018 to 2021, the consistency of this pathway is no longer stable. Under the condition of multiple co-existence, this reflects the challenges of coordination with the system and continuous adaptability.

In 2016, the H4 pathway covered the development of service capabilities in multiple provinces including Shandong, Sichuan, Anhui, and Hunan. It covers the most cases of that year, making it the most prominent path for the development of high primary healthcare capabilities. However, in the following years, the development landscape underwent significant changes. The applicability of H4 has significantly decreased, and only Anhui Province remained under this pathway in 2017. From 2016 to 2021, the consistency of Configuration H4 always exceeded 0.9, showing its strong impact on improving the ability of primary healthcare services. However, the overall trend shows consistent fluctuations over the years. Talent shortage is an obvious weakness. High-intensity financial investment and high-level policy support can partially solve the talent gap in the short term and promote the application of high-tech innovation to meet public demand. However, the lack of talents will continue to restrict the continuous change of technology and the long-term development of services. Furthermore, in environments with lower levels of economic development, the sustainability of fiscal investment and the actual effectiveness of policy implementation may also be affected.

Overall, the inter-group analysis revealed the characteristics as “temporal stability and intertemporal heterogeneity of configuration paths.” Inter-group consistency analysis showed that the four high-level configuration paths (H1–H4) maintained a high level of consistency (pooled consistency all above 0.92) between 2016 and 2021, indicating that all four paths are stable configurations that explain the high level of primary healthcare service capacity. However, significant differences exist in the temporal stability of each path: Pathways H3 and H4 exhibited the strongest temporal stability, with smaller annual consistency fluctuations (0.926–0.996) and a pooled consistency of 0.969, suggesting that the “internal and external integrated synergy” and “policy-demand dual-driven” paths are relatively universal high-level paths. The consistency between paths H1 and H2 declined significantly in the later period (2020–2021), especially for path H2, which dropped from 0.964 in 2018 to 0.821 in 2021. This indicates that the stability of the “demand-technology compensation” path is affected by time factors, and its effectiveness may change with the policy or technology diffusion stage.

#### 4.3.2 Spatial analysis of service capability in high-level primary healthcare institutions

To further analyze the spatial differences in paths, this study conducted an intra-group consistency adjustment distance for each pathway. From a spatial perspective, the pathway of high PHIs service capability mainly serves the economically developed regions in central and eastern China. Beijing, Guangdong, Zhejiang, Jiangsu and Sichuan are typical examples. Most provinces have maintained a high level of service capability before 2021. In addition, the evolution of the service capability pathway in high-level PHIs shows that each province follows different development trajectories at different stages. With the continuous strengthening of government support for primary healthcare services, the development has increasingly turned to H2 and H3 pathways.

**Figure 9:**
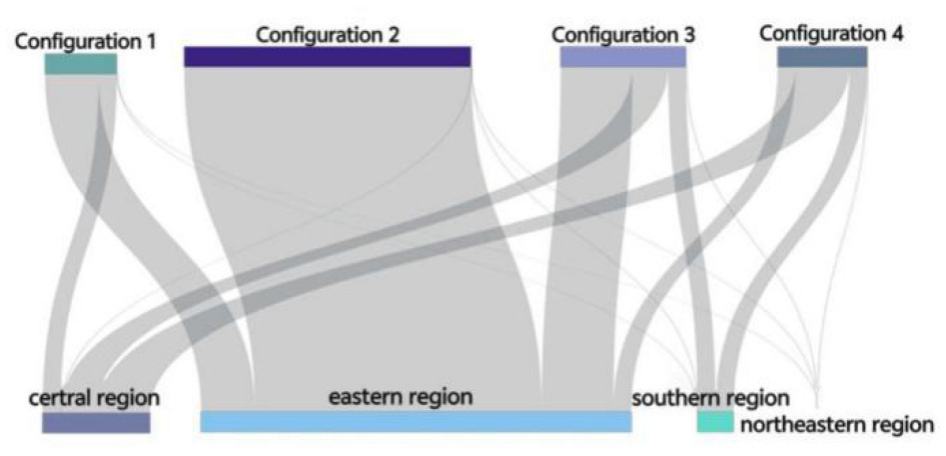
Geographic distribution of the configurations.

The four conditional configurations show significant regional differences in Figure 9. H1 mainly exists in the eastern and central regions. H2 is concentrated in the eastern region. H3 is primarily distributed in the eastern region, followed by the central and western regions. H4 is mainly located in the central region, with secondary occurrences in the eastern and western regions. This regional difference stems from the interaction of many factors, including the level of economic development, the strength of policy support, the distribution of medical resources and the adaptability of health policy.

Within-group consistency analysis revealed significant differences in the stability of provincial affiliation with the four paths over time: The first category comprises path-specific provinces, including Anhui, Jiangsu, Shandong, Guangdong, and Sichuan, which actually exhibit high-level configurations in the map data. These provinces all have within-group consistency close to 1.000 for specific paths, and their coverage distribution closely matches their actual configurations. The second category consists of mixed-type provinces, such as Beijing, Shanghai, and Tianjin, which have some coverage of multiple paths, reflecting their path switching characteristics over time. The third category comprises marginal provinces, including Heilongjiang, Jilin, Inner Mongolia, Liaoning, Qinghai, and Shaanxi. These provinces generally have low within-group consistency (some below 0.8), dispersed coverage distribution, and no clearly dominant path, indicating that they failed to stably belong to any high-level configuration during the sample period, consistent with their characteristic of consistently appearing in the map data as “NO data” or only in a few years.

(1) H2 Path: The “Robust” Pattern and “Policy-Technology” Synergistic Lock-in in the East

The H2 path is spatially highly concentrated in the eastern coastal areas, including Beijing, Tianjin, Shanghai, Jiangsu, Zhejiang, and, in its early stages, Guangdong. It has formed a stable contiguous belt in the Yangtze River Delta and the Beijing-Tianjin-Hebei region, showing a trend of spreading outwards, but has not yet extended to the central and western regions.

The underlying mechanism of this stable pattern is the synergy between policy and technology. The eastern region boasts a developed economy (environmental advantages), strong policy support (environmental advantages), and active technological innovation (technological advantages). Although its internal organizational elements (such as the healthcare workforce density) may not be comprehensively superior, it effectively substitutes for organizational weaknesses through technological means—for example, Zhejiang uses the digital economy to promote telemedicine, and Beijing establishes a smart pharmacy system. This model exhibits path dependence, thus achieving self-reinforcement and stable continuation in the eastern region. Policy synergy and technological spillover effects from regional integration (such as Shanghai's radiation to surrounding areas) further solidify this pattern.

(2) The H1 Path: The Double-Edged Sword Effect of Technology Compensation

The H1 path spatially only appeared in Guangdong (2017, 2018, 2020) and Anhui (2019), exhibiting a highly volatile, “flash-like” characteristic. Guangdong, as a hub of technological innovation, switched between H2 and H1; Anhui, however, quickly disappeared after a brief attempt at technology-driven development with policy support.

The underlying mechanism of this volatility stems from the double-edged sword effect of technology compensation. The H1 path relies on technological innovation (technological factors) to functionally substitute for the healthcare workforce shortages (organizational disadvantages), but the effectiveness of this compensation mechanism depends on whether technology can continuously iterate and accurately match grassroots needs. From a dynamic capability perspective, H1 regions, after “perceiving” needs and technological opportunities, can quickly “capture” external resources. However, their insufficient “reconstruction” capacity and failure to deeply embed technology applications into organizational systems and institutional frameworks ultimately led to the path's interruption.

(3) The H4 Path: A “Transitional” Path for Populous Provinces

The H4 path was widely distributed in populous provinces such as Anhui, Shandong, Hunan, and Sichuan in 2016. However, most were subsequently replaced by H3 or H1, with only Shandong successfully upgrading, while Hunan and Anhui dropped out of the high-level ranks.

This divergence reflects the essential characteristic of H4 as a “transitional” path. It represents the initial stage of capacity building achieved by leveraging strong public demand (environmental factors) and policy support (environmental factors) when internal organizational foundations are weak. However, if external drivers are not transformed into internal capacity accumulation (organizational factor improvement), it is highly susceptible to dropping out of the high-level ranks. Shandong and Sichuan achieved a leap to H3 by continuously investing resources to address internal shortcomings; while Hunan and Anhui experienced interruptions. Furthermore, the disappearance of H3 in Hubei and the brief existence of H4 in Hunan collectively suggest that a high-level configuration is not a one-time achievement. It requires continuous resource guarantees and dynamic capabilities. Once resources are lost or capabilities decline, the original coordination or compensation mechanisms may collapse.

(4) The H3 Path: The “Ideal” Model of Resource-Rich Areas and the Leap from H4

The H3 path exhibits a spatially “point-like” characteristic, appearing only in Shandong (most years from 2017 to 2021), Hubei (2016–2017), and Sichuan (2020). It is noteworthy that these cases largely evolved from the H4 path. Shandong transitioned from H4 in 2016 and maintained this position for a long period, while Sichuan also transitioned from H4.

The underlying logic of this leap is the evolution “from policy-demand compensation to systemic collaboration.” Initially, H4 regions (such as Shandong and Sichuan) primarily relied on strong public demand (environmental factors) and policy support (environmental factors) to drive service capability, while facing internal shortcomings such as the healthcare workforce shortages and insufficient financial investment (organizational disadvantages). With continuous resource investment, organizational factors gradually accumulated. Shandong strengthened its primary healthcare workforce through the “county-hired, township-employed” policy; Sichuan filled the healthcare workforce gap through the “Primary Health Personnel Capacity Building Plan.” Simultaneously, continuous investment in technology (technical factors) ultimately achieved balanced development of the three factors, leaping to the H3 path. This process transforms external policy dividends and demand pressures into internal capability accumulation, and leverages technology to achieve overall systemic upgrading. However, this leap is highly dependent on long-term, stable resource investment. Once policy priorities shift or financial investment decreases, the collaborative mechanism may collapse, as evidenced by the disappearance of H3 in Hubei.

Therefore, regional strategies must be adapted to the actual capability of local PHIs. To reduce disparities and promote sustainable development, differentiated approaches are essential: optimize collaboration mechanisms and resource integration in the eastern region, address talent and infrastructure gaps in the central region, and strengthen financial and policy support to foster endogenous growth in the western region. These targeted measures will help to balance space progress and sustainably improve primary healthcare capability.

### 4.4 Robustness tests

According to the principle that “results are considered robust if they remain substantively unchanged after minor adjustments to QCA procedures,” this study conducts a series of sensitivity analyses to examine the robustness of the empirical results. The core research conclusions under examination include: (1) the identification of four primary causal pathways leading to the outcome, (2) the consistent presence of X5 and X7 as core conditions across most configurations. The results of the sensitivity analysis are shown in Table 8.

**Table 8:** The results of the sensitivity analysis.

Test type	Adjust	Intermediate solution	Consistancy	PRI	Coverage	Result change	Conclusion
Original results		~X2^*^X3^*^X4^*^X5^*^X7+~X1^*^~X3^*^X4^*^X5^*^X6^*^X7+X1^*^X2^*^X3^*^X5^*^X6^*^X7 +~X2^*^X3^*^~X4^*^X5^*^X6^*^X7 -> Y	0.935	0.861	0.503	————————————————————	
Calibration threshold	95/50/5 → 90/50/10	~X2^*^X3^*^X5^*^X6^*^X7 +~X1^*^~X2^*^X4^*^X5^*^X6^*^X7	0.928	0.868	0.444	The number of paths has been reduced from four to two; however, these two paths can be separately mapped to the four original paths, with X5 and X7 remaining as high-frequency conditions for configuration.	Core conditions remain stable
	95/50/5 → 75/50/25	~X1^*^~X2^*^X3^*^X4^*^X5^**^X7+X1^*^X3^*^~X4^*^X5^*^X6+X1^*^X2^*^X4^*^X5^*^X6^*^X7+~X2^*^X3^*^~X4^*^X5^*^X6^*^	0.94	0.919	0.324	The configuration expression has changed, but the core conditions X5 and X7 still appear	Partially robust
Consistency threshold	0.9 → 0.8	~X1^*^~X2^*^X3^*^X4^*^X5^*^X7 + ~X1^*^~X3^*^X4^*^X5^*^X6^*^X7 + X1^*^X2^*^X3^*^X5^*^X6^*^X7 + ~X2^*^X3^*^~X4^*^X5^*^X6^*^X7 -> Y	0.935	0.861	0.503	The configuration expression is completely consistent	Highly robust
PRI threshold	0.8 → 0.7	M1:X1^*^X3^*^~X4^*^X5^*^X6+~X2^*^X3^*^~X4^*^X5^*^X6+X1^*^X2^*^X4^*^X5^*^X6^*^X7 + (~X1^*^~X2^*^X3^*^X4^*^X5^*^X7 + ~X1^*^X2^*^X4^*^X5^*^~X6^*^X7 + ~X1^*^~X3^*^X4^*^X5^*^X6^*^X7) -> Y	0.909	0.82	0.61	The configuration expression has been augmented, yet the original four paths remain preserved (in parentheses)	The core path remains robust
		M2:X1^*^X3^*^~X4^*^X5^*^X6+~X2^*^X3^*^~X4^*^X5^*^X6+X1^*^X2^*^X4^*^X5^*^X6^*^X7 + (~X1^*^~X2^*^X4^*^X5^*^X6^*^X7 + ~X1^*^X2^*^~X3^*^X4^*^X5^*^X7 + ~X1^*^X3^*^X4^*^X5^*^~X6^*^X7)-> Y	0.907	0.815	0.612		
Frequency	2 → 3	M1: ~X1^*^~X2^*^X3^*^X5^*^X6^*^X7 + ~X1^*^~X3^*^X4^*^X5^*^X6^*^X7 + X1^*^X2^*^X3^*^X4^*^X5^*^X6^*^X7 -> Y	0.938	0.864	0.437	The configuration expression has been streamlined, yet the core conditions X5, X6, and X7 remain present.	The core path remains robust

First, sensitivity to calibration parameters was tested. For this purpose, the original membership anchors for fuzzy-set calibration were adjusted. Specifically, the full membership and full non-membership thresholds were reset to the 90th and 10th percentiles, and then to the 75th and 25th percentiles, respectively. The full membership and full non-membership thresholds are reset to 90% and 10%, and 75% and 25%, respectively. After re-running the QCA procedure, the number of configurational solutions changes slightly. After re-running the QCA procedure, the number of configurational solutions changed slightly. When the thresholds were adjusted to 90/50/10, the four original pathways were consolidated into two. However, these two paths could be directly mapped back to the original four, with X5 and X7 remaining as high-frequency core conditions. When the thresholds were adjusted to 75/50/25, the expression of the configurations changed, but X5 and X7 still appeared consistently. Thus, the core conditions remain largely consistent with the original solution, indicating that the results are insensitive to calibration parameters. In addition, for each calibration group, we re-performed the necessity and sufficiency analyses. In accordance with the best practices of QCA (95), we focused on the intermediate solutions during the sufficiency analysis, as they only include the theoretically defensible counterfactual configurations and are the main basis for substantive explanations (96).

Subsequently, the consistency threshold was varied to test whether the results were driven by an arbitrary cutoff. The sufficiency consistency threshold was raised to 0.80, and the sufficiency analysis was re-performed. The results show that the configuration expression remained completely identical to the original solution, with all four core pathways and their constituent conditions preserved. This confirms that the findings are highly robust and not driven by arbitrary threshold choices.

Additionally, the Proportional Reduction in Inconsistency (PRI) threshold was lowered from 0.8 to 0.7. While this adjustment generated more configurational expressions, the original four pathways were still preserved within the solution. Although some minor pathways emerged, the core causal pathways remained intact, further supporting the stability and reliability of the core conclusions.

Finally, the frequency threshold was increased from 2 to 3 to exclude configurations that might be driven by outlier cases. The resulting solution was streamlined from four paths to three. Crucially, the core conditions—particularly X5, X6, and X7—remained present in the reduced set. Although overall coverage decreased moderately, the retention of these core conditions reinforces the reliability of the core findings.

In summary, these sensitivity tests demonstrate that the core conclusions regarding the primary pathways and the necessity of X5 and X7 remained stable, which passed standard robustness checks, exhibiting strong stability and credibility across methodological variations.

## 5 Research conclusions and implications

### 5.1 Research conclusions

The dynamic QCA of 30 provinces in China revealed the path to improve the service capability of PHIs from 2016 to 2021, compared to previous studies that focused on data from a single year (21). The main findings show that: first, although there is no single condition to prove that high service capability is necessary, as previous studies have concluded (21), the necessity of human resources and economic development has been increasing over time. It may be that with the standardized construction of PHIs in China, the infrastructure and institutional allocation are gradually improved, and the impact of human resources and economic development is more prominent. Second, the adequacy analysis identified four solution configurations integrated into three types of paths: the demand technology compensation model, the leverage path of government science and technology collaboration, and the integrated configuration path of internal organization and external environment.

The details of the different paths are as follows: Paths H1 and H4 indicate that when organizational factors are at a disadvantage (such as the healthcare workforce shortages), capacity compensation can be achieved through functional substitution by technological factors and pressure-driven environmental factors. The characteristic of the H2 path is that, under conditions where organizational elements are not optimal, policy guides the application of technology, which in turn optimizes organizational processes. The H3 path is the result of high-level collaboration among the three elements of TOE (Total Needs, Energy, and Environment), forming an evolutionary dynamic capability of “perceiving needs - integrating resources - reconstructing models.”

### 5.2 Policy implications

Based on the above path analysis, this study proposes feasible policy solutions from the TOE-DC theoretical framework. This study argues that improving primary healthcare service capability is essentially a process of organizations building dynamic capabilities of “perception-capture-reconstruction” under the constraints of technology, organization, and environment. Therefore, policy intervention should not be a static allocation of resources, but rather a differentiated capacity governance approach.

#### 5.2.1 Policy intervention based on the demand-technology compensation path

For this path characteristic, the primary task of policy intervention is to establish an institutionalized compensation mechanism for technology introduction and ensure its sustainability. Specifically, the government should promote private investment through tax incentives, targeted subsidies and simplified approval. For example, including intelligent assisted diagnosis and treatment systems in basic public health service funding; and provincial-level unified procurement of AI-assisted diagnostic systems for township health centers.

Secondly, differentiated resource acquisition channels need to be designed. For H1-category regions, a “tax credit for technology procurement” should be implemented to encourage institutions to procure services such as telemedicine and AI diagnosis through market-based methods. For H4-category regions, a “provincial-county technology transfer payment” system should be established, with the provincial government procuring technology resources and allocating them to the county as needed.

Finally, a transitional mechanism from compensation to endogenous development needs to be constructed. Simultaneously, the talent retention mechanism characterized by competitive compensation and career development systems should be strengthened. For example, the “county-hired, township-employed” policy can be overlaid with a “remote mentoring” module, allowing county-level doctors to guide township doctors on daily ward rounds through a remote system, achieving simultaneous technology compensation and talent accumulation.

#### 5.2.2 Policy intervention in the government-technology collaborative upgrading path

Based on this, the primary focus of policy intervention is to promote the construction of a policy-technology collaborative governance platform. For example, promoting data sharing among the Health Commission, Healthcare Security Administration, Medical Products Administration, and Big Data Administration to establish a “Regional Primary Healthcare Collaborative Governance Platform” to achieve precise matching of policy and technological resources. Secondly, a policy incubation mechanism for innovative applications needs to be established, such as setting up “pilot programs for innovative applications of primary healthcare technology.” For innovative projects, “first-time trial and first-use” policy support should be provided, such as exemption from certain approval processes.

#### 5.2.3 Policy intervention for integrated internal and external collaborative paths

Given the characteristics of this path, the primary task of policy intervention is to establish an early warning and intervention mechanism for capability maintenance. For example, constructing an “early warning indicator system for the degradation of primary healthcare service capabilities,” including indicators such as the healthcare workforce loss rate, frequency of technology use, and fluctuations in financial investment, setting thresholds to trigger provincial-level intervention. Secondly, support should be provided to H4 regions to facilitate their transition to H3 status through bundled investment of “technology + talent + finance.” Finally, a regional collaborative capacity radiation mechanism should be promoted, encouraging H3 regions to establish “medical consortium alliances” with surrounding H2 and H4 regions. Capacity spillover may be achieved through a model of “technology output + talent training + management trusteeship.”

### 5.3 Limitations

In terms of conceptual definition, we adopted the concept of acctualized capability (including medical service output and efficiency). Compared to potential capability, this definition has the following limitations. First, this measurement is easily affected by external environmental factors, primarily because the quantity of healthcare service output depends not only on the institution itself but also on local population structure, health insurance policies, and even transportation accessibility. Second, this measurement neglects the quality of healthcare services by focusing solely on their quantity and efficiency, such as misdiagnosis rate and patient satisfaction. Although introducing efficiency indicators such as “average length of stay” has mitigated this problem, the issue remains. Finally, due to limited access to micro-level clinical quality data, indicators such as employee skill mix, service scope, and equipment availability are not reflected in output metrics, especially in underserved areas where demand may be suppressed. Furthermore, in poverty-stricken areas where demand is severely suppressed, output may underestimate the actual readiness.

Regarding research data, this study uses provincial panel data, which, while reflecting inter-provincial differences and temporal variations, may mask heterogeneity at the city and county levels within the province. Secondly, there are gaps in the sample coverage. Hong Kong Special Administrative Region, Macao Special Administrative Region, Taiwan region, and Tibet Autonomous Region were excluded due to missing data or inconsistent statistical methods. These regions have unique characteristics in terms of resource endowment, governance structure, and service system. Their primary healthcare development paths may rely more on central government transfer payments and external resources than on the paths identified in this study.

In terms of research methods, although QCA, as a set-theoretic approach, excels at identifying the necessary and sufficient relationship between combinations of conditions and outcomes, its causal inference capabilities have inherent limitations. For example, this study found that path H4 may transition to H3, but the current analysis cannot reveal the specific process and triggering conditions of this transition. Furthermore, dynamic QCA presents a challenge in balancing the number of cases with the duration of time. This study included 31 cases across 6 years, totaling 186 “case-year” observation points, which met the analytical requirements. However, some paths are supported by only a few cases (e.g., H1 only appeared in Guangdong and Anhui for several years), and the stability of its temporal evolution trend needs to be verified by more cases.

## Data Availability

The data of this article comes from the public database. The original contributions presented in this study are included in the article/Supplementary material. Further inquiries can be directed to the corresponding authors.
