# Supplementary material for: Pathways for enhancing service capability of primary healthcare institutions: a dynamic qualitative comparative analysis
Source: Front Public Health. 2026 Apr 23;14:1791172. doi: 10.3389/fpubh.2026.1791172 (PMC13149366; doi:10.3389/fpubh.2026.1791172)
Supplement: Supplementary file 1 [file Table_1.docx]

**Supplementary table 1.** Variables Weights in the Rank-Sum Ratio Method (%)

| Variant dimension | 2016 | 2017 | 2018 | 2019 | 2020 | 2021 |
| --- | --- | --- | --- | --- | --- | --- |
| Number of outpatient visits in primary healthcare institutions | 10.247 | 9.968 | 10.036 | 9.836 | 8.822 | 8.811 |
| Number of admissions to primary healthare institution | 12.819 | 12.148 | 12.322 | 12.957 | 12.601 | 12.334 |
| Number of home health visits by community health center(stations) | 15.753 | 16.618 | 16.694 | 15.949 | 14.006 | 10.676 |
| Bed occupancy rate in community health enters | 3.628 | 3.622 | 4.345 | 5.752 | 6.036 | 4.435 |
| Bed occupancy rate in township hospital | 3.772 | 4.455 | 5.014 | 3.245 | 3.43 | 3.621 |
| Average length of stay(ALOS)in community health centers | 21.633 | 22.233 | 21.19 | 21.872 | 27.886 | 32.357 |
| Average length of stay(ALOS)in township hospitals | 4.795 | 4.57 | 3.661 | 3.868 | 4.961 | 4.584 |
| Average daily outpatient visits per physician in community health center | 13.256 | 12.692 | 13.656 | 11.804 | 7.742 | 8.159 |

**Supplementary table 2.** Weighted Rank-Sum Ratio (WRSR) Ranking of Provinces

|  | 2016 | 2017 | 2018 | 2019 | 2020 | 2021 |
| --- | --- | --- | --- | --- | --- | --- |
| Tianjin | 16 | 15 | 17 | 17 | 21 | 18 |
| Beijing | 15 | 19 | 18 | 15 | 16 | 17 |
| Hebei | 11 | 14 | 14 | 14 | 15 | 19 |
| Shanxi | 25 | 25 | 25 | 25 | 23 | 26 |
| Inner Mongolia | 28 | 28 | 27 | 29 | 29 | 29 |
| Liaoning | 21 | 20 | 21 | 22 | 24 | 24 |
| Jilin | 30 | 30 | 30 | 30 | 28 | 27 |
| Heilongjiang | 27 | 27 | 28 | 27 | 30 | 30 |
| Shanghai | 1 | 1 | 1 | 1 | 1 | 1 |
| Jiangsu | 3 | 2 | 4 | 4 | 5 | 7 |
| Zhejiang | 5 | 5 | 5 | 5 | 7 | 4 |
| Anhui | 14 | 9 | 9 | 8 | 8 | 11 |
| Fujian | 19 | 18 | 15 | 16 | 14 | 14 |
| Jiangxi | 18 | 16 | 16 | 19 | 17 | 16 |
| Shandong | 6 | 7 | 7 | 7 | 6 | 3 |
| Henan | 8 | 6 | 6 | 6 | 4 | 6 |
| Hubei | 7 | 8 | 8 | 9 | 10 | 10 |
| Hunan | 9 | 10 | 10 | 10 | 9 | 8 |
| Guangdong | 2 | 3 | 2 | 3 | 3 | 5 |
| Guangxi | 12 | 13 | 12 | 12 | 12 | 12 |
| Hainan | 22 | 23 | 26 | 24 | 25 | 23 |
| Chongqing | 13 | 11 | 11 | 11 | 11 | 9 |
| Sichuan | 4 | 4 | 3 | 2 | 2 | 2 |
| Guizhou | 26 | 26 | 24 | 20 | 19 | 15 |
| Yunnan | 10 | 12 | 13 | 13 | 13 | 13 |
| Shaanxi | 23 | 21 | 20 | 21 | 22 | 25 |
| Gansu | 24 | 24 | 22 | 23 | 20 | 22 |
| Qinghai | 29 | 29 | 29 | 28 | 27 | 28 |
| Ningxia | 20 | 22 | 23 | 26 | 26 | 21 |
| Xinjiang | 17 | 17 | 19 | 18 | 18 | 20 |

**Supplementary table 3.** Comprehensive Measurement Results of Antecedent Variables​

| **province** | **year** | **Infrastructure** | **Talent pool** | **Financial investment** | **Economic development** | **Public demand** | **Policy support** | **Technology innovation** |
| --- | --- | --- | --- | --- | --- | --- | --- | --- |
| Tianjin | 2016 | 0.165528569 | 0.073934132 | 0.05493549 | 0.383876289 | 0.173766 | 0.0001 | 0.385005253 |
| Beijing | 2016 | 0.116515182 | 0.507768508 | 0.062113983 | 0.655280611 | 0.256679527 | 22 | 0.747394415 |
| Hebei | 2016 | 0.686526954 | 0.216640178 | 0.090562407 | 0.110899191 | 0.414172185 | 15 | 0.088441411 |
| Shanxi | 2016 | 0.750000474 | 0.318727112 | 0.087743448 | 0.082458953 | 0.206922505 | 13 | 0.063958733 |
| Inner Mongolia | 2016 | 0.626481731 | 0.336499012 | 0.063072965 | 0.220333223 | 0.182629357 | 19 | 0.051140465 |
| Liaoning | 2016 | 0.502252352 | 0.16200518 | 0.067135339 | 0.211280494 | 0.31784432 | 26 | 0.139560338 |
| Jilin | 2016 | 0.481183528 | 0.305115572 | 0.076300372 | 0.119987666 | 0.20064864 | 15 | 0.07023637 |
| Heilongjiang | 2016 | 0.37496419 | 0.10919565 | 0.066367976 | 0.091276711 | 0.250068544 | 19 | 0.079160386 |
| Shanghai | 2016 | 0.139964446 | 0.277486328 | 0.055369753 | 0.671866285 | 0.297321531 | 7 | 0.691598718 |
| Jiangsu | 2016 | 0.298972642 | 0.329762338 | 0.071405816 | 0.386685418 | 0.490648333 | 25 | 0.511962369 |
| Zhejiang | 2016 | 0.220204022 | 0.397545223 | 0.077777427 | 0.429641069 | 0.371768914 | 7 | 0.367227278 |
| Anhui | 2016 | 0.323939415 | 0.11731349 | 0.086931803 | 0.133585853 | 0.328972856 | 8 | 0.167615503 |
| Fujian | 2016 | 0.424871961 | 0.246257396 | 0.088314544 | 0.301801781 | 0.21684531 | 28 | 0.205965275 |
| Jiangxi | 2016 | 0.612028509 | 0.14982805 | 0.09501451 | 0.124302576 | 0.214212664 | 31 | 0.092130578 |
| Shandong | 2016 | 0.564452083 | 0.372927096 | 0.090253689 | 0.233902863 | 0.56492298 | 24 | 0.257251858 |
| Henan | 2016 | 0.539291664 | 0.226600659 | 0.104378473 | 0.107690806 | 0.532287969 | 20 | 0.12114842 |
| Hubei | 2016 | 0.590394914 | 0.426579163 | 0.091686413 | 0.195745291 | 0.363852469 | 13 | 0.169878589 |
| Hunan | 2016 | 0.780450961 | 0.3158092 | 0.086173878 | 0.156214261 | 0.367941608 | 17 | 0.145224475 |
| Guangdong | 2016 | 0.209214084 | 0.164023584 | 0.083431689 | 0.321142125 | 0.649829089 | 25 | 0.438903472 |
| Guangxi | 2016 | 0.564888799 | 0.330733004 | 0.105405588 | 0.071334314 | 0.247644614 | 38 | 0.079505748 |
| Hainan | 2016 | 0.306385173 | 0.260459715 | 0.08294345 | 0.138160594 | 0.049250459 | 7 | 0.17108518 |
| Chongqing | 2016 | 0.639603504 | 0.295233133 | 0.082757552 | 0.202607311 | 0.19670635 | 1 | 0.213960728 |
| Sichuan | 2016 | 0.79808036 | 0.339401375 | 0.09642285 | 0.107559723 | 0.456054881 | 19 | 0.165226743 |
| Guizhou | 2016 | 0.549851198 | 0.15832881 | 0.092087482 | 0.028013871 | 0.170650102 | 13 | 0.108122577 |
| Yunnan | 2016 | 0.416918334 | 0.086179124 | 0.093045034 | 0.062253111 | 0.244749885 | 6 | 0.052907859 |
| Shaanxi | 2016 | 0.577156417 | 0.246651891 | 0.086950975 | 0.141145151 | 0.255799146 | 6 | 0.18679331 |
| Gansu | 2016 | 0.710549096 | 0.284781079 | 0.086745206 | 0 | 0.143220757 | 7 | 0.078458911 |
| Qinghai | 2016 | 0.634459581 | 0.171932526 | 0.067589192 | 0.085767849 | 0.076529593 | 7 | 0.039806152 |
| Ningxia | 2016 | 0.278550919 | 0.093406336 | 0.065386516 | 0.107989352 | 0.079585993 | 4 | 0.100469333 |
| Xinjiang | 2016 | 0.584342743 | 0.262573544 | 0.061965807 | 0.101522623 | 0.158951876 | 5 | 0.049612073 |
| Tianjin | 2017 | 0.181656336 | 0.182455596 | 0.055475333 | 0.430733469 | 0.208047818 | 2 | 0.328825635 |
| Beijing | 2017 | 0.120482479 | 0.602676595 | 0.062695893 | 0.722299398 | 0.300233521 | 30 | 0.765270022 |
| Hebei | 2017 | 0.716713179 | 0.280578819 | 0.091140773 | 0.14119153 | 0.43278409 | 20 | 0.065938415 |
| Shanxi | 2017 | 0.767888442 | 0.354329873 | 0.085544215 | 0.128821101 | 0.219691683 | 4 | 0.034323807 |
| Inner Mongolia | 2017 | 0.656731558 | 0.395861469 | 0.071409492 | 0.25575476 | 0.190708452 | 23 | 0.041981065 |
| Liaoning | 2017 | 0.508022144 | 0.207000101 | 0.068989757 | 0.241287682 | 0.325661682 | 29 | 0.097499164 |
| Jilin | 2017 | 0.502320336 | 0.343451402 | 0.074943903 | 0.145472235 | 0.211861913 | 3 | 0.065802421 |
| Heilongjiang | 2017 | 0.405912419 | 0.170812539 | 0.064030355 | 0.114775579 | 0.256117378 | 8 | 0.052386349 |
| Shanghai | 2017 | 0.138204659 | 0.338595838 | 0.054610592 | 0.738152329 | 0.28561943 | 14 | 0.552159144 |
| Jiangsu | 2017 | 0.338413125 | 0.414273428 | 0.07433554 | 0.434665977 | 0.498522973 | 30 | 0.449909247 |
| Zhejiang | 2017 | 0.223027065 | 0.450463826 | 0.077575721 | 0.482137277 | 0.395656243 | 7 | 0.340864576 |
| Anhui | 2017 | 0.356290593 | 0.143377202 | 0.096350469 | 0.172769843 | 0.334564246 | 18 | 0.136520613 |
| Fujian | 2017 | 0.421002787 | 0.271762491 | 0.089758014 | 0.348343338 | 0.224526418 | 14 | 0.182075922 |
| Jiangxi | 2017 | 0.649752977 | 0.163140661 | 0.096369538 | 0.160024348 | 0.226146458 | 33 | 0.076342803 |
| Shandong | 2017 | 0.576234837 | 0.41286769 | 0.089569472 | 0.268246959 | 0.582417574 | 26 | 0.208251062 |
| Henan | 2017 | 0.563494656 | 0.249907429 | 0.101838958 | 0.142851078 | 0.545478396 | 14 | 0.08716503 |
| Hubei | 2017 | 0.594389724 | 0.447831237 | 0.090378842 | 0.234256552 | 0.395330017 | 9 | 0.154151179 |
| Hunan | 2017 | 0.791565004 | 0.351341915 | 0.085303062 | 0.192515363 | 0.393809297 | 4 | 0.119390684 |
| Guangdong | 2017 | 0.213749537 | 0.210436407 | 0.086953399 | 0.365106688 | 0.679496888 | 17 | 0.446678771 |
| Guangxi | 2017 | 0.574662393 | 0.359921235 | 0.104370945 | 0.103139905 | 0.266851035 | 60 | 0.054111332 |
| Hainan | 2017 | 0.325976552 | 0.285215506 | 0.088208204 | 0.171910992 | 0.057933317 | 7 | 0.118734738 |
| Chongqing | 2017 | 0.633199106 | 0.301058984 | 0.081588366 | 0.241109941 | 0.211045875 | 2 | 0.176662703 |
| Sichuan | 2017 | 0.825310502 | 0.38210017 | 0.095627711 | 0.147698836 | 0.472593286 | 13 | 0.134271432 |
| Guizhou | 2017 | 0.563236328 | 0.219982758 | 0.094570864 | 0.066362011 | 0.185492276 | 10 | 0.067742613 |
| Yunnan | 2017 | 0.447529966 | 0.206965912 | 0.095745295 | 0.099150835 | 0.260233886 | 14 | 0.046459682 |
| Shaanxi | 2017 | 0.587440072 | 0.291344338 | 0.086541187 | 0.178523113 | 0.274755954 | 7 | 0.144814036 |
| Gansu | 2017 | 0.744475738 | 0.322000489 | 0.087530716 | 0.024368846 | 0.154200686 | 2 | 0.047101922 |
| Qinghai | 2017 | 0.665371623 | 0.290481025 | 0.081813073 | 0.117187652 | 0.086055356 | 5 | 0.045859621 |
| Ningxia | 2017 | 0.291824597 | 0.124105638 | 0.071373417 | 0.147088536 | 0.088019838 | 8 | 0.08922502 |
| Xinjiang | 2017 | 0.595588584 | 0.264227229 | 0.057514815 | 0.13985501 | 0.174818391 | 4 | 0.036844745 |
| Tianjin | 2018 | 0.186197855 | 0.261695939 | 0.062117326 | 0.47262936 | 0.234775131 | 0.0001 | 0.328298284 |
| Beijing | 2018 | 0.127552894 | 0.726372299 | 0.065595207 | 0.794735157 | 0.336937265 | 11 | 0.822774737 |
| Hebei | 2018 | 0.76258899 | 0.351090197 | 0.089478541 | 0.171467051 | 0.447860208 | 3 | 0.092102304 |
| Shanxi | 2018 | 0.767583606 | 0.390145745 | 0.083799613 | 0.161874387 | 0.246310409 | 1 | 0.058353634 |
| Inner Mongolia | 2018 | 0.680635199 | 0.426610285 | 0.065326009 | 0.292716457 | 0.20914177 | 19 | 0.057775576 |
| Liaoning | 2018 | 0.53141691 | 0.24926289 | 0.065687222 | 0.275283904 | 0.34979438 | 10 | 0.11050795 |
| Jilin | 2018 | 0.548106023 | 0.476241066 | 0.074208556 | 0.168520153 | 0.228629567 | 1 | 0.086172484 |
| Heilongjiang | 2018 | 0.413826825 | 0.19198465 | 0.064360934 | 0.141410533 | 0.295856461 | 4 | 0.056540245 |
| Shanghai | 2018 | 0.136720286 | 0.396092657 | 0.056291415 | 0.808828503 | 0.332077731 | 46 | 0.599760341 |
| Jiangsu | 2018 | 0.358548408 | 0.530630661 | 0.072513908 | 0.480697124 | 0.549406774 | 46 | 0.459985994 |
| Zhejiang | 2018 | 0.231085288 | 0.499504754 | 0.072564786 | 0.537159601 | 0.436872276 | 4 | 0.390171674 |
| Anhui | 2018 | 0.368152756 | 0.17596216 | 0.095417786 | 0.217348192 | 0.344237932 | 7 | 0.154054908 |
| Fujian | 2018 | 0.427260131 | 0.342716302 | 0.091398372 | 0.396487486 | 0.239340852 | 11 | 0.212866069 |
| Jiangxi | 2018 | 0.643567483 | 0.173754576 | 0.103302679 | 0.200933439 | 0.238255866 | 2 | 0.094634225 |
| Shandong | 2018 | 0.593929357 | 0.503387732 | 0.087630285 | 0.301209195 | 0.598856039 | 17 | 0.223861559 |
| Henan | 2018 | 0.580959559 | 0.288323948 | 0.100778608 | 0.179143006 | 0.578954944 | 9 | 0.112780926 |
| Hubei | 2018 | 0.624449541 | 0.46761728 | 0.079321932 | 0.27578932 | 0.402864328 | 3 | 0.176804073 |
| Hunan | 2018 | 0.784575811 | 0.35766657 | 0.08384127 | 0.227184543 | 0.42156445 | 4 | 0.139792604 |
| Guangdong | 2018 | 0.21958324 | 0.278689995 | 0.089483548 | 0.40670303 | 0.710390033 | 4 | 0.524378297 |
| Guangxi | 2018 | 0.581279393 | 0.393504009 | 0.102908446 | 0.134755174 | 0.297384588 | 60 | 0.074180469 |
| Hainan | 2018 | 0.321851717 | 0.339238765 | 0.085413587 | 0.206079082 | 0.071719912 | 7 | 0.13008385 |
| Chongqing | 2018 | 0.658477836 | 0.404799502 | 0.082095156 | 0.275852128 | 0.230541121 | 1 | 0.197241005 |
| Sichuan | 2018 | 0.843131733 | 0.432616911 | 0.09074324 | 0.18753193 | 0.498709437 | 3 | 0.149556545 |
| Guizhou | 2018 | 0.573076412 | 0.26489066 | 0.095791382 | 0.103351711 | 0.209328651 | 0.0001 | 0.08648486 |
| Yunnan | 2018 | 0.46945618 | 0.270254841 | 0.094718874 | 0.137319204 | 0.274757098 | 5 | 0.067828331 |
| Shaanxi | 2018 | 0.585475457 | 0.350583828 | 0.085868015 | 0.215549451 | 0.280587111 | 4 | 0.166081701 |
| Gansu | 2018 | 0.739843653 | 0.333599655 | 0.083115293 | 0.056706915 | 0.187266979 | 2 | 0.055521915 |
| Qinghai | 2018 | 0.681757377 | 0.333171474 | 0.085952059 | 0.153189643 | 0.110004942 | 3 | 0.086301742 |
| Ningxia | 2018 | 0.311127781 | 0.24177711 | 0.074380224 | 0.180316042 | 0.1053289 | 11 | 0.093838038 |
| Xinjiang | 2018 | 0.587145123 | 0.260728739 | 0.057085856 | 0.175774921 | 0.189372266 | 1 | 0.046414351 |
| Tianjin | 2019 | 0.1831307 | 0.322650006 | 0.055645708 | 0.513749099 | 0.265820507 | 6 | 0.336501044 |
| Beijing | 2019 | 0.134978369 | 0.853604258 | 0.072137729 | 0.864529538 | 0.382519698 | 7 | 0.878823073 |
| Hebei | 2019 | 0.756394612 | 0.418397213 | 0.083652263 | 0.207095969 | 0.464555734 | 8 | 0.111623448 |
| Shanxi | 2019 | 0.767885787 | 0.464870627 | 0.07783882 | 0.192026906 | 0.264270568 | 0.0001 | 0.077192153 |
| Inner Mongolia | 2019 | 0.676350951 | 0.498709662 | 0.063161279 | 0.32740923 | 0.234350815 | 6 | 0.066331331 |
| Liaoning | 2019 | 0.507544406 | 0.270543678 | 0.063452444 | 0.307976129 | 0.366436126 | 22 | 0.129934847 |
| Jilin | 2019 | 0.535302207 | 0.516867327 | 0.071614524 | 0.196911594 | 0.242606172 | 4 | 0.080532141 |
| Heilongjiang | 2019 | 0.466460825 | 0.244538747 | 0.062738948 | 0.17010081 | 0.313772038 | 7 | 0.06762613 |
| Shanghai | 2019 | 0.139552188 | 0.454725714 | 0.060328048 | 0.874094116 | 0.345619937 | 14 | 0.654091559 |
| Jiangsu | 2019 | 0.383752081 | 0.638974027 | 0.072056818 | 0.525343098 | 0.565384751 | 20 | 0.469220558 |
| Zhejiang | 2019 | 0.246139569 | 0.572283381 | 0.073172964 | 0.590591162 | 0.448560078 | 14 | 0.430159816 |
| Anhui | 2019 | 0.387223014 | 0.263671874 | 0.092984246 | 0.256265099 | 0.371213158 | 6 | 0.173962696 |
| Fujian | 2019 | 0.432179365 | 0.403655497 | 0.092116276 | 0.441916066 | 0.267828622 | 4 | 0.230913345 |
| Jiangxi | 2019 | 0.651913694 | 0.234500607 | 0.098795954 | 0.237902613 | 0.264395085 | 1 | 0.130613889 |
| Shandong | 2019 | 0.602805366 | 0.592762609 | 0.084924617 | 0.335799667 | 0.618967169 | 16 | 0.230183399 |
| Henan | 2019 | 0.59309997 | 0.325001081 | 0.097086462 | 0.211449408 | 0.601037907 | 3 | 0.130362344 |
| Hubei | 2019 | 0.630166952 | 0.468436089 | 0.075508675 | 0.315310012 | 0.435125277 | 4 | 0.201494756 |
| Hunan | 2019 | 0.812078159 | 0.580860057 | 0.082343218 | 0.268767208 | 0.44698612 | 8 | 0.165269817 |
| Guangdong | 2019 | 0.226724554 | 0.323515379 | 0.091317707 | 0.451753155 | 0.742480737 | 7 | 0.552615649 |
| Guangxi | 2019 | 0.607377814 | 0.460073577 | 0.096614914 | 0.16632383 | 0.323956579 | 41 | 0.090496257 |
| Hainan | 2019 | 0.316521296 | 0.396424175 | 0.091359087 | 0.240302984 | 0.0781051 | 3 | 0.155426198 |
| Chongqing | 2019 | 0.685734043 | 0.512704363 | 0.079060499 | 0.316728668 | 0.257950819 | 3 | 0.225248347 |
| Sichuan | 2019 | 0.868071074 | 0.532213602 | 0.091153315 | 0.223862287 | 0.536297744 | 6 | 0.177791209 |
| Guizhou | 2019 | 0.592943624 | 0.350357118 | 0.089898029 | 0.138069331 | 0.229514304 | 1 | 0.114802554 |
| Yunnan | 2019 | 0.482687387 | 0.407700326 | 0.089880637 | 0.175909177 | 0.288479566 | 3 | 0.082842816 |
| Shaanxi | 2019 | 0.587142613 | 0.468404904 | 0.081540329 | 0.250021122 | 0.303678772 | 2 | 0.193533651 |
| Gansu | 2019 | 0.735173644 | 0.400629071 | 0.082601984 | 0.086910618 | 0.19140358 | 3 | 0.070274301 |
| Qinghai | 2019 | 0.700946359 | 0.409276854 | 0.079536613 | 0.183862598 | 0.125252051 | 11 | 0.101350109 |
| Ningxia | 2019 | 0.308968713 | 0.320092242 | 0.074039311 | 0.211449277 | 0.125564212 | 9 | 0.126359672 |
| Xinjiang | 2019 | 0.6299457 | 0.281415452 | 0.056882809 | 0.200315919 | 0.204510812 | 2 | 0.043561525 |
| Tianjin | 2020 | 0.170592092 | 0.379190661 | 0.055687245 | 0.52870551 | 0.231955194 | 7 | 0.300288451 |
| Beijing | 2020 | 0.144801527 | 0.912526042 | 0.085107459 | 0.885117227 | 0.360229956 | 16 | 0.854117589 |
| Hebei | 2020 | 0.740046333 | 0.472823666 | 0.090578413 | 0.22737539 | 0.464781654 | 4 | 0.099053374 |
| Shanxi | 2020 | 0.743589689 | 0.498094582 | 0.084829002 | 0.215497333 | 0.267165029 | 7 | 0.060561534 |
| Inner Mongolia | 2020 | 0.675708225 | 0.560559301 | 0.071164822 | 0.338703272 | 0.212442365 | 14 | 0.030026516 |
| Liaoning | 2020 | 0.503243382 | 0.296835798 | 0.068757617 | 0.319638969 | 0.352387798 | 18 | 0.132734942 |
| Jilin | 2020 | 0.614174183 | 0.734066984 | 0.072737493 | 0.219760897 | 0.225948159 | 15 | 0.072607176 |
| Heilongjiang | 2020 | 0.440513715 | 0.292212926 | 0.073620814 | 0.18209147 | 0.266642875 | 9 | 0.041683955 |
| Shanghai | 2020 | 0.141794786 | 0.548742178 | 0.067273834 | 0.908048659 | 0.329161902 | 3 | 0.543401422 |
| Jiangsu | 2020 | 0.399452766 | 0.703753667 | 0.073637124 | 0.553334383 | 0.551288513 | 23 | 0.416768702 |
| Zhejiang | 2020 | 0.245680315 | 0.622427439 | 0.083202655 | 0.620909979 | 0.437184535 | 9 | 0.392768905 |
| Anhui | 2020 | 0.463618912 | 0.468719817 | 0.101908186 | 0.279473238 | 0.377617989 | 6 | 0.185489319 |
| Fujian | 2020 | 0.445027348 | 0.467145158 | 0.1000671 | 0.462856725 | 0.276621841 | 6 | 0.1729365 |
| Jiangxi | 2020 | 0.65634202 | 0.294369879 | 0.096246973 | 0.264002652 | 0.281527934 | 1 | 0.127010906 |
| Shandong | 2020 | 0.602170659 | 0.641833531 | 0.09306967 | 0.35419971 | 0.631713327 | 19 | 0.241677698 |
| Henan | 2020 | 0.62614611 | 0.42709898 | 0.104639403 | 0.222292412 | 0.590988775 | 4 | 0.121066519 |
| Hubei | 2020 | 0.654291752 | 0.517351657 | 0.120777507 | 0.304024509 | 0.379577741 | 5 | 0.192512866 |
| Hunan | 2020 | 0.815786875 | 0.558489124 | 0.087779197 | 0.293662639 | 0.454434335 | 5 | 0.147847956 |
| Guangdong | 2020 | 0.236048052 | 0.368106155 | 0.101715986 | 0.476521874 | 0.740649498 | 9 | 0.544731535 |
| Guangxi | 2020 | 0.625441065 | 0.563028871 | 0.101115468 | 0.18516333 | 0.318548996 | 44 | 0.069771873 |
| Hainan | 2020 | 0.418314833 | 0.570099534 | 0.111581477 | 0.258080418 | 0.090146357 | 4 | 0.121538416 |
| Chongqing | 2020 | 0.679428272 | 0.595254486 | 0.088772873 | 0.345815795 | 0.276374939 | 3 | 0.182612035 |
| Sichuan | 2020 | 0.864054259 | 0.5876646 | 0.09202271 | 0.250437669 | 0.534730367 | 11 | 0.17237842 |
| Guizhou | 2020 | 0.62357946 | 0.442155053 | 0.098555623 | 0.162719129 | 0.229540894 | 4 | 0.088132421 |
| Yunnan | 2020 | 0.502393326 | 0.527308492 | 0.10199856 | 0.197802947 | 0.303248892 | 3 | 0.04822034 |
| Shaanxi | 2020 | 0.578128623 | 0.496717328 | 0.085858773 | 0.267847279 | 0.31418959 | 0.0001 | 0.198564974 |
| Gansu | 2020 | 0.742627737 | 0.433638239 | 0.088927319 | 0.10514425 | 0.183646848 | 9 | 0.053452931 |
| Qinghai | 2020 | 0.639618141 | 0.443699313 | 0.089034788 | 0.202064475 | 0.123458754 | 9 | 0.064046941 |
| Ningxia | 2020 | 0.318756055 | 0.438414636 | 0.080095382 | 0.233309968 | 0.123520178 | 5 | 0.09295099 |
| Xinjiang | 2020 | 0.554788241 | 0.262312737 | 0.085242429 | 0.20856435 | 0.195009364 | 1 | 0.03509172 |
| Tianjin | 2021 | 0.179583768 | 0.501731187 | 0.057915021 | 0.586790556 | 0.392247072 | 16 | 0.321203353 |
| Beijing | 2021 | 0.148527479 | 1 | 0.087808392 | 0.967163232 | 0.38324187 | 22 | 0.940589638 |
| Hebei | 2021 | 0.752160191 | 0.541302907 | 0.096459058 | 0.270412042 | 0.492591089 | 3 | 0.138431832 |
| Shanxi | 2021 | 0.746639379 | 0.559028286 | 0.082585968 | 0.280275913 | 0.274602824 | 4 | 0.087776971 |
| Inner Mongolia | 2021 | 0.692032805 | 0.61895485 | 0.069228963 | 0.400687793 | 0.257774636 | 1 | 0.058435769 |
| Liaoning | 2021 | 0.499456162 | 0.366423176 | 0.067908784 | 0.362135021 | 0.368856205 | 0.0001 | 0.141492664 |
| Jilin | 2021 | 0.627249533 | 0.770427484 | 0.075664622 | 0.255774425 | 0.257020651 | 4 | 0.12842733 |
| Heilongjiang | 2021 | 0.465814489 | 0.32281388 | 0.076845172 | 0.223875116 | 0.308564692 | 3 | 0.092179726 |
| Shanghai | 2021 | 0.148368523 | 0.660655654 | 0.075095542 | 0.989166781 | 0.412158704 | 5 | 0.60154456 |
| Jiangsu | 2021 | 0.412457084 | 0.742838338 | 0.081157964 | 0.619121657 | 0.596479283 | 20 | 0.450710577 |
| Zhejiang | 2021 | 0.242101425 | 0.685040715 | 0.082439746 | 0.696595982 | 0.494384246 | 10 | 0.443448457 |
| Anhui | 2021 | 0.47559146 | 0.541516331 | 0.096429348 | 0.327753694 | 0.401187432 | 3 | 0.226208571 |
| Fujian | 2021 | 0.449455876 | 0.516128407 | 0.10252809 | 0.52019587 | 0.296218201 | 13 | 0.206841572 |
| Jiangxi | 2021 | 0.67660399 | 0.331538767 | 0.094893397 | 0.31581281 | 0.306612772 | 0.0001 | 0.159168562 |
| Shandong | 2021 | 0.616428057 | 0.673602803 | 0.093289941 | 0.405334371 | 0.641950865 | 19 | 0.261730673 |
| Henan | 2021 | 0.689858289 | 0.506454185 | 0.104104641 | 0.255184281 | 0.604068121 | 6 | 0.13689312 |
| Hubei | 2021 | 0.668055315 | 0.583212496 | 0.089029415 | 0.362146077 | 0.430679438 | 5 | 0.223318279 |
| Hunan | 2021 | 0.818880622 | 0.562391506 | 0.088873942 | 0.337787464 | 0.461792384 | 0.0001 | 0.186785014 |
| Guangdong | 2021 | 0.247838759 | 0.429278674 | 0.101775579 | 0.536992028 | 0.765775445 | 2 | 0.593455199 |
| Guangxi | 2021 | 0.658862647 | 0.602792982 | 0.10569978 | 0.228988451 | 0.340347685 | 24 | 0.113735075 |
| Hainan | 2021 | 0.438677179 | 0.677236045 | 0.098494955 | 0.309084695 | 0.117644424 | 10 | 0.135264839 |
| Chongqing | 2021 | 0.701807786 | 0.668123849 | 0.088462191 | 0.396106684 | 0.298565891 | 1 | 0.198833577 |
| Sichuan | 2021 | 0.843789687 | 0.656594562 | 0.093096368 | 0.295699923 | 0.550881635 | 6 | 0.212112328 |
| Guizhou | 2021 | 0.628404418 | 0.471859991 | 0.096971204 | 0.200437009 | 0.238902565 | 4 | 0.122151718 |
| Yunnan | 2021 | 0.511893121 | 0.567084278 | 0.109428792 | 0.240326476 | 0.316527017 | 0.0001 | 0.082792342 |
| Shaanxi | 2021 | 0.582680229 | 0.515604944 | 0.093211319 | 0.316864052 | 0.332423603 | 2 | 0.220801157 |
| Gansu | 2021 | 0.744889286 | 0.47542508 | 0.096806991 | 0.145638246 | 0.204339125 | 2 | 0.090821874 |
| Qinghai | 2021 | 0.652292693 | 0.431380108 | 0.095081207 | 0.241146072 | 0.119819579 | 12 | 0.078085507 |
| Ningxia | 2021 | 0.307272327 | 0.461324987 | 0.077540987 | 0.280021437 | 0.145371998 | 4 | 0.10658967 |
| Xinjiang | 2021 | 0.610893087 | 0.251317717 | 0.082765008 | 0.258960669 | 0.232179344 | 1 | 0.055435301 |

Note：Due to computational requirements, a value of 0.0001 was assigned to results where the comprehensive measure value was 0.

**Supplementary table 4.** Data Calibration Results of Dynamic QCA

| province | year | X1 | X2 | X3 | X4 | X5 | X6 | X7 | Y |
| --- | --- | --- | --- | --- | --- | --- | --- | --- | --- |
| Tianjin | 2016 | 0.057258 | 0.016798 | 0.038738 | 0.706979 | 0.131887 | 0.032085 | 0.829806 | 0.545053 |
| Beijing | 2016 | 0.041589 | 0.767544 | 0.078556 | 0.926348 | 0.351109 | 0.871705 | 0.979738 | 0.577335 |
| Hebei | 2016 | 0.820755 | 0.095984 | 0.69239 | 0.077315 | 0.754395 | 0.735349 | 0.176045 | 0.653399 |
| Shanxi | 2016 | 0.915188 | 0.281754 | 0.584388 | 0.046114 | 0.201583 | 0.682752 | 0.086351 | 0.249973 |
| Inner Mongolia | 2016 | 0.670521 | 0.329982 | 0.086115 | 0.410235 | 0.148225 | 0.82243 | 0.058082 | 0.131425 |
| Liaoning | 2016 | 0.379589 | 0.050112 | 0.125869 | 0.368636 | 0.550191 | 0.91887 | 0.507584 | 0.400689 |
| Jilin | 2016 | 0.346191 | 0.247866 | 0.272631 | 0.090822 | 0.186556 | 0.735349 | 0.104342 | 0.046469 |
| Heilongjiang | 2016 | 0.203511 | 0.026131 | 0.117317 | 0.054223 | 0.328396 | 0.82243 | 0.135571 | 0.143567 |
| Shanghai | 2016 | 0.048495 | 0.187898 | 0.040461 | 0.932943 | 0.501318 | 0.5 | 0.971399 | 0.97619 |
| Jiangsu | 2016 | 0.131725 | 0.311202 | 0.183589 | 0.710507 | 0.864501 | 0.908823 | 0.915909 | 0.942366 |
| Zhejiang | 2016 | 0.081201 | 0.514729 | 0.304251 | 0.761183 | 0.67192 | 0.5 | 0.813316 | 0.877567 |
| Anhui | 2016 | 0.152578 | 0.02891 | 0.551157 | 0.115 | 0.576351 | 0.531892 | 0.551803 | 0.615179 |
| Fujian | 2016 | 0.264614 | 0.134294 | 0.607337 | 0.594221 | 0.22716 | 0.93599 | 0.610818 | 0.496845 |
| Jiangxi | 2016 | 0.62606 | 0.043191 | 0.825548 | 0.097952 | 0.220158 | 0.955459 | 0.194587 | 0.51488 |
| Shandong | 2016 | 0.483855 | 0.439812 | 0.681308 | 0.47488 | 0.928452 | 0.89767 | 0.684698 | 0.869577 |
| Henan | 2016 | 0.440976 | 0.107633 | 0.957601 | 0.073003 | 0.904755 | 0.840324 | 0.388471 | 0.864769 |
| Hubei | 2016 | 0.555565 | 0.588569 | 0.730846 | 0.301847 | 0.655027 | 0.682752 | 0.555344 | 0.868529 |
| Hunan | 2016 | 0.94213 | 0.274257 | 0.519701 | 0.167559 | 0.663805 | 0.782009 | 0.516544 | 0.688602 |
| Guangdong | 2016 | 0.07575 | 0.051356 | 0.441043 | 0.622248 | 0.966905 | 0.908823 | 0.872749 | 0.943812 |
| Guangxi | 2016 | 0.484603 | 0.313872 | 0.96404 | 0.037522 | 0.320259 | 0.981293 | 0.136925 | 0.636554 |
| Hainan | 2016 | 0.13765 | 0.156871 | 0.42852 | 0.124316 | 0.022055 | 0.5 | 0.557229 | 0.338182 |
| Chongqing | 2016 | 0.708427 | 0.225039 | 0.423775 | 0.330526 | 0.177564 | 0.051168 | 0.622781 | 0.623993 |
| Sichuan | 2016 | 0.953825 | 0.338248 | 0.856861 | 0.072831 | 0.820911 | 0.82243 | 0.54806 | 0.921042 |
| Guizhou | 2016 | 0.458902 | 0.047918 | 0.743809 | 0.016588 | 0.126517 | 0.682752 | 0.291585 | 0.175443 |
| Yunnan | 2016 | 0.254134 | 0.019593 | 0.773062 | 0.03167 | 0.310683 | 0.380675 | 0.061388 | 0.66407 |
| Shaanxi | 2016 | 0.511096 | 0.134882 | 0.551948 | 0.130737 | 0.348042 | 0.380675 | 0.581607 | 0.276241 |
| Gansu | 2016 | 0.863645 | 0.202564 | 0.543441 | 0.009717 | 0.086882 | 0.5 | 0.132855 | 0.265645 |
| Qinghai | 2016 | 0.693873 | 0.056519 | 0.131175 | 0.049012 | 0.033118 | 0.5 | 0.040556 | 0.064266 |
| Ningxia | 2016 | 0.116512 | 0.021452 | 0.107117 | 0.073394 | 0.03465 | 0.188459 | 0.241831 | 0.427983 |
| Xinjiang | 2016 | 0.535303 | 0.160483 | 0.077444 | 0.06533 | 0.108001 | 0.274209 | 0.055358 | 0.518023 |
| Tianjin | 2017 | 0.063531 | 0.064147 | 0.040891 | 0.762389 | 0.204372 | 0.080659 | 0.77357 | 0.499069 |
| Beijing | 2017 | 0.042687 | 0.897732 | 0.083067 | 0.94977 | 0.508276 | 0.949696 | 0.981867 | 0.428156 |
| Hebei | 2017 | 0.873155 | 0.194013 | 0.71256 | 0.130839 | 0.785851 | 0.840324 | 0.091699 | 0.532466 |
| Shanxi | 2017 | 0.932155 | 0.382252 | 0.495896 | 0.105949 | 0.234906 | 0.188459 | 0.034016 | 0.165671 |
| Inner Mongolia | 2017 | 0.753817 | 0.510395 | 0.183646 | 0.525305 | 0.164541 | 0.885326 | 0.043471 | 0.082942 |
| Liaoning | 2017 | 0.388954 | 0.085797 | 0.148753 | 0.503323 | 0.568605 | 0.94323 | 0.224154 | 0.305848 |
| Jilin | 2017 | 0.379699 | 0.349949 | 0.245473 | 0.140544 | 0.214037 | 0.124909 | 0.091322 | 0.045167 |
| Heilongjiang | 2017 | 0.24009 | 0.055759 | 0.094312 | 0.082835 | 0.349149 | 0.531892 | 0.060395 | 0.113348 |
| Shanghai | 2017 | 0.047941 | 0.335944 | 0.037494 | 0.954177 | 0.457398 | 0.709754 | 0.933548 | 0.965723 |
| Jiangsu | 2017 | 0.165864 | 0.557562 | 0.233902 | 0.766695 | 0.873079 | 0.949696 | 0.880288 | 0.954979 |
| Zhejiang | 2017 | 0.082657 | 0.646599 | 0.299813 | 0.814353 | 0.72015 | 0.5 | 0.786642 | 0.863319 |
| Anhui | 2017 | 0.183533 | 0.039904 | 0.855373 | 0.217065 | 0.589345 | 0.803001 | 0.502774 | 0.656954 |
| Fujian | 2017 | 0.259481 | 0.176971 | 0.663082 | 0.66029 | 0.248478 | 0.709754 | 0.574322 | 0.429922 |
| Jiangxi | 2017 | 0.735912 | 0.050808 | 0.855766 | 0.17809 | 0.253142 | 0.965152 | 0.124941 | 0.49449 |
| Shandong | 2017 | 0.507986 | 0.553987 | 0.656016 | 0.54421 | 0.938795 | 0.91887 | 0.614252 | 0.808428 |
| Henan | 2017 | 0.482215 | 0.13982 | 0.936639 | 0.134532 | 0.915079 | 0.709754 | 0.169961 | 0.833609 |
| Hubei | 2017 | 0.568843 | 0.640377 | 0.685826 | 0.476586 | 0.719522 | 0.563526 | 0.53064 | 0.784469 |
| Hunan | 2017 | 0.949791 | 0.373261 | 0.489604 | 0.288849 | 0.716579 | 0.188459 | 0.374654 | 0.656788 |
| Guangdong | 2017 | 0.077958 | 0.08931 | 0.552048 | 0.682778 | 0.974872 | 0.782009 | 0.878116 | 0.935063 |
| Guangxi | 2017 | 0.502677 | 0.399291 | 0.95755 | 0.067266 | 0.387378 | 0.998854 | 0.06374 | 0.558019 |
| Hainan | 2017 | 0.154394 | 0.203464 | 0.603097 | 0.214256 | 0.025114 | 0.5 | 0.369549 | 0.216155 |
| Chongqing | 2017 | 0.690245 | 0.238312 | 0.394284 | 0.503053 | 0.211942 | 0.080659 | 0.565923 | 0.590698 |
| Sichuan | 2017 | 0.967572 | 0.468917 | 0.839801 | 0.145827 | 0.842986 | 0.682752 | 0.495863 | 0.920687 |
| Guizhou | 2017 | 0.481772 | 0.099762 | 0.814626 | 0.0342 | 0.153848 | 0.59465 | 0.096829 | 0.154504 |
| Yunnan | 2017 | 0.295944 | 0.085762 | 0.842424 | 0.062584 | 0.363613 | 0.709754 | 0.050116 | 0.577436 |
| Shaanxi | 2017 | 0.545691 | 0.216476 | 0.53498 | 0.236568 | 0.416479 | 0.5 | 0.515895 | 0.263573 |
| Gansu | 2017 | 0.909215 | 0.290311 | 0.57574 | 0.015476 | 0.101186 | 0.080659 | 0.051144 | 0.203965 |
| Qinghai | 2017 | 0.774826 | 0.214607 | 0.3999 | 0.086449 | 0.038123 | 0.274209 | 0.049173 | 0.049207 |
| Ningxia | 2017 | 0.126217 | 0.031454 | 0.183082 | 0.144363 | 0.039242 | 0.531892 | 0.179863 | 0.252085 |
| Xinjiang | 2017 | 0.57281 | 0.163356 | 0.050106 | 0.127927 | 0.133744 | 0.188459 | 0.036886 | 0.462162 |
| Tianjin | 2018 | 0.06541 | 0.158975 | 0.078582 | 0.805449 | 0.278938 | 0.032085 | 0.772985 | 0.476832 |
| Beijing | 2018 | 0.044714 | 0.969131 | 0.109219 | 0.967083 | 0.594823 | 0.625033 | 0.98733 | 0.453415 |
| Hebei | 2018 | 0.927487 | 0.372508 | 0.652583 | 0.212814 | 0.809104 | 0.124909 | 0.19444 | 0.543274 |
| Shanxi | 2018 | 0.931895 | 0.494626 | 0.450532 | 0.183387 | 0.315826 | 0.051168 | 0.072712 | 0.12879 |
| Inner Mongolia | 2018 | 0.808749 | 0.588647 | 0.106515 | 0.580827 | 0.20711 | 0.82243 | 0.071424 | 0.084789 |
| Liaoning | 2018 | 0.427704 | 0.13883 | 0.110157 | 0.554808 | 0.624065 | 0.59465 | 0.308268 | 0.289899 |
| Jilin | 2018 | 0.45593 | 0.704681 | 0.231535 | 0.203423 | 0.260401 | 0.051168 | 0.165347 | 0.053006 |
| Heilongjiang | 2018 | 0.250135 | 0.071871 | 0.097301 | 0.131321 | 0.496502 | 0.188459 | 0.068742 | 0.056261 |
| Shanghai | 2018 | 0.047478 | 0.51099 | 0.044366 | 0.969706 | 0.583581 | 0.993186 | 0.949973 | 0.960845 |
| Jiangsu | 2018 | 0.185865 | 0.806906 | 0.201563 | 0.813025 | 0.917952 | 0.993186 | 0.88685 | 0.942471 |
| Zhejiang | 2018 | 0.086947 | 0.75201 | 0.202419 | 0.859758 | 0.792354 | 0.188459 | 0.834376 | 0.875904 |
| Anhui | 2018 | 0.196042 | 0.059334 | 0.835031 | 0.396343 | 0.611524 | 0.5 | 0.530488 | 0.700151 |
| Fujian | 2018 | 0.267814 | 0.347811 | 0.721285 | 0.722617 | 0.293221 | 0.625033 | 0.621152 | 0.503631 |
| Jiangxi | 2018 | 0.719361 | 0.057776 | 0.949681 | 0.323402 | 0.289788 | 0.080659 | 0.207993 | 0.483501 |
| Shandong | 2018 | 0.567318 | 0.759395 | 0.579794 | 0.593351 | 0.947225 | 0.782009 | 0.63739 | 0.824892 |
| Henan | 2018 | 0.52392 | 0.209989 | 0.925288 | 0.23874 | 0.936866 | 0.563526 | 0.324645 | 0.831765 |
| Hubei | 2018 | 0.66443 | 0.685862 | 0.339412 | 0.555567 | 0.733823 | 0.124909 | 0.566143 | 0.78642 |
| Hunan | 2018 | 0.945093 | 0.392388 | 0.451608 | 0.44263 | 0.767251 | 0.188459 | 0.507952 | 0.686831 |
| Guangdong | 2018 | 0.080883 | 0.19026 | 0.652772 | 0.7349 | 0.981176 | 0.188459 | 0.921769 | 0.952341 |
| Guangxi | 2018 | 0.524998 | 0.504325 | 0.946442 | 0.117322 | 0.501469 | 0.998854 | 0.117276 | 0.592142 |
| Hainan | 2018 | 0.150736 | 0.337782 | 0.492487 | 0.345548 | 0.030838 | 0.5 | 0.461065 | 0.107802 |
| Chongqing | 2018 | 0.758168 | 0.533368 | 0.406986 | 0.555661 | 0.26608 | 0.051168 | 0.59761 | 0.619418 |
| Sichuan | 2018 | 0.974331 | 0.603544 | 0.698781 | 0.269461 | 0.873276 | 0.124909 | 0.523389 | 0.943613 |
| Guizhou | 2018 | 0.49864 | 0.16452 | 0.843442 | 0.067523 | 0.207581 | 0.032085 | 0.166788 | 0.205461 |
| Yunnan | 2018 | 0.328214 | 0.174178 | 0.818327 | 0.122555 | 0.416483 | 0.274209 | 0.097079 | 0.57485 |
| Shaanxi | 2018 | 0.539106 | 0.370994 | 0.506948 | 0.388051 | 0.438337 | 0.188459 | 0.5494 | 0.300443 |
| Gansu | 2018 | 0.903917 | 0.321828 | 0.432918 | 0.028541 | 0.157421 | 0.080659 | 0.066602 | 0.228033 |
| Qinghai | 2018 | 0.811082 | 0.320633 | 0.510454 | 0.159557 | 0.054107 | 0.124909 | 0.165942 | 0.055966 |
| Ningxia | 2018 | 0.141558 | 0.127765 | 0.234739 | 0.242888 | 0.050555 | 0.625033 | 0.203657 | 0.209037 |
| Xinjiang | 2018 | 0.544703 | 0.157327 | 0.048018 | 0.227104 | 0.161746 | 0.051168 | 0.050044 | 0.342039 |
| Tianjin | 2019 | 0.064136 | 0.292028 | 0.041595 | 0.841691 | 0.383638 | 0.380675 | 0.781968 | 0.450444 |
| Beijing | 2019 | 0.046941 | 0.991485 | 0.195317 | 0.978223 | 0.69416 | 0.5 | 0.991081 | 0.500416 |
| Hebei | 2019 | 0.921655 | 0.568015 | 0.446727 | 0.350009 | 0.832547 | 0.531892 | 0.316248 | 0.510979 |
| Shanxi | 2019 | 0.932153 | 0.679733 | 0.305609 | 0.286912 | 0.378039 | 0.032085 | 0.128068 | 0.122503 |
| Inner Mongolia | 2019 | 0.799639 | 0.750479 | 0.086843 | 0.631167 | 0.277632 | 0.380675 | 0.092795 | 0.05139 |
| Liaoning | 2019 | 0.388175 | 0.17471 | 0.089284 | 0.603245 | 0.660586 | 0.871705 | 0.459831 | 0.227879 |
| Jilin | 2019 | 0.43424 | 0.783849 | 0.186876 | 0.306622 | 0.303694 | 0.188459 | 0.141016 | 0.049931 |
| Heilongjiang | 2019 | 0.323699 | 0.131757 | 0.08341 | 0.208421 | 0.54054 | 0.5 | 0.09649 | 0.060759 |
| Shanghai | 2019 | 0.048364 | 0.656566 | 0.066079 | 0.979429 | 0.614658 | 0.709754 | 0.964009 | 0.96066 |
| Jiangsu | 2019 | 0.213458 | 0.92732 | 0.193993 | 0.850864 | 0.928745 | 0.840324 | 0.892585 | 0.918597 |
| Zhejiang | 2019 | 0.095502 | 0.865198 | 0.212862 | 0.894575 | 0.810135 | 0.709754 | 0.866473 | 0.876044 |
| Anhui | 2019 | 0.217483 | 0.162386 | 0.771277 | 0.526079 | 0.670748 | 0.380675 | 0.561719 | 0.792905 |
| Fujian | 2019 | 0.274483 | 0.530433 | 0.744724 | 0.774492 | 0.390938 | 0.188459 | 0.647644 | 0.494374 |
| Jiangxi | 2019 | 0.741544 | 0.117739 | 0.898944 | 0.494197 | 0.378488 | 0.051168 | 0.465455 | 0.381248 |
| Shandong | 2019 | 0.596472 | 0.887967 | 0.479738 | 0.642969 | 0.956049 | 0.759451 | 0.646588 | 0.804495 |
| Henan | 2019 | 0.564566 | 0.298289 | 0.869917 | 0.369397 | 0.948258 | 0.124909 | 0.463371 | 0.846664 |
| Hubei | 2019 | 0.681422 | 0.687676 | 0.256555 | 0.613873 | 0.789593 | 0.188459 | 0.604069 | 0.791349 |
| Hunan | 2019 | 0.961472 | 0.875175 | 0.41325 | 0.544995 | 0.807811 | 0.531892 | 0.548127 | 0.714041 |
| Guangdong | 2019 | 0.084601 | 0.294323 | 0.71857 | 0.784772 | 0.986079 | 0.5 | 0.933727 | 0.947518 |
| Guangxi | 2019 | 0.611243 | 0.66888 | 0.860748 | 0.196626 | 0.564603 | 0.987172 | 0.186196 | 0.607988 |
| Hainan | 2019 | 0.146114 | 0.511844 | 0.719965 | 0.501825 | 0.033899 | 0.124909 | 0.53265 | 0.149277 |
| Chongqing | 2019 | 0.819174 | 0.776494 | 0.333321 | 0.615917 | 0.355558 | 0.124909 | 0.639416 | 0.673983 |
| Sichuan | 2019 | 0.981536 | 0.809435 | 0.712988 | 0.426843 | 0.908006 | 0.380675 | 0.567677 | 0.955817 |
| Guizhou | 2019 | 0.564047 | 0.370317 | 0.668283 | 0.124124 | 0.26302 | 0.051168 | 0.339582 | 0.286263 |
| Yunnan | 2019 | 0.34853 | 0.540798 | 0.667639 | 0.22756 | 0.468291 | 0.124909 | 0.150601 | 0.581492 |
| Shaanxi | 2019 | 0.544695 | 0.687607 | 0.393087 | 0.5166 | 0.516504 | 0.080659 | 0.591953 | 0.283565 |
| Gansu | 2019 | 0.898298 | 0.522661 | 0.419814 | 0.050053 | 0.16601 | 0.124909 | 0.10446 | 0.207003 |
| Qinghai | 2019 | 0.84764 | 0.544829 | 0.344454 | 0.255722 | 0.067384 | 0.625033 | 0.247252 | 0.053832 |
| Ningxia | 2019 | 0.139768 | 0.285304 | 0.228407 | 0.369396 | 0.067685 | 0.563526 | 0.430422 | 0.116442 |
| Xinjiang | 2019 | 0.680773 | 0.195694 | 0.047058 | 0.320795 | 0.195702 | 0.080659 | 0.045714 | 0.401998 |
| Tianjin | 2020 | 0.059162 | 0.459653 | 0.041768 | 0.853441 | 0.270332 | 0.5 | 0.740374 | 0.212478 |
| Beijing | 2020 | 0.050049 | 0.995342 | 0.484503 | 0.980738 | 0.647161 | 0.759451 | 0.989587 | 0.366449 |
| Hebei | 2020 | 0.904155 | 0.697302 | 0.692959 | 0.443541 | 0.832848 | 0.188459 | 0.233287 | 0.451728 |
| Shanxi | 2020 | 0.908222 | 0.749291 | 0.477247 | 0.387812 | 0.38852 | 0.5 | 0.07783 | 0.14439 |
| Inner Mongolia | 2020 | 0.798245 | 0.85048 | 0.179848 | 0.647014 | 0.215537 | 0.709754 | 0.029613 | 0.044992 |
| Liaoning | 2020 | 0.381191 | 0.228637 | 0.145711 | 0.620096 | 0.629862 | 0.803001 | 0.483072 | 0.126284 |
| Jilin | 2020 | 0.632818 | 0.971416 | 0.205345 | 0.407559 | 0.252568 | 0.735349 | 0.111958 | 0.050004 |
| Heilongjiang | 2020 | 0.286014 | 0.218368 | 0.220799 | 0.249258 | 0.386622 | 0.563526 | 0.043062 | 0.029303 |
| Shanghai | 2020 | 0.049077 | 0.834329 | 0.127469 | 0.983204 | 0.576793 | 0.124909 | 0.930024 | 0.965204 |
| Jiangsu | 2020 | 0.232099 | 0.961343 | 0.221093 | 0.871209 | 0.919296 | 0.885326 | 0.856356 | 0.849734 |
| Zhejiang | 2020 | 0.095231 | 0.91496 | 0.435158 | 0.910747 | 0.792844 | 0.563526 | 0.836636 | 0.792844 |
| Anhui | 2020 | 0.319446 | 0.688304 | 0.937321 | 0.561093 | 0.684124 | 0.380675 | 0.579596 | 0.767056 |
| Fujian | 2020 | 0.292379 | 0.684813 | 0.916652 | 0.795963 | 0.423442 | 0.380675 | 0.560119 | 0.585465 |
| Jiangxi | 2020 | 0.75284 | 0.223117 | 0.853223 | 0.537798 | 0.441889 | 0.051168 | 0.435749 | 0.348012 |
| Shandong | 2020 | 0.594408 | 0.92928 | 0.773783 | 0.668235 | 0.960893 | 0.82243 | 0.663034 | 0.831775 |
| Henan | 2020 | 0.669519 | 0.589865 | 0.959334 | 0.419432 | 0.943337 | 0.188459 | 0.387823 | 0.854829 |
| Hubei | 2020 | 0.747652 | 0.784693 | 0.997141 | 0.597477 | 0.688158 | 0.274209 | 0.590391 | 0.709414 |
| Hunan | 2020 | 0.963285 | 0.847747 | 0.585837 | 0.582228 | 0.818622 | 0.274209 | 0.52069 | 0.76548 |
| Guangdong | 2020 | 0.089688 | 0.424668 | 0.93541 | 0.809133 | 0.985837 | 0.563526 | 0.93057 | 0.886641 |
| Guangxi | 2020 | 0.667409 | 0.853686 | 0.929083 | 0.260539 | 0.551857 | 0.99122 | 0.102905 | 0.614765 |
| Hainan | 2020 | 0.255954 | 0.862553 | 0.986869 | 0.528831 | 0.040489 | 0.188459 | 0.391562 | 0.109071 |
| Chongqing | 2020 | 0.806215 | 0.890495 | 0.625423 | 0.656832 | 0.422518 | 0.124909 | 0.575151 | 0.693726 |
| Sichuan | 2020 | 0.980526 | 0.882635 | 0.741743 | 0.517233 | 0.906747 | 0.625033 | 0.559248 | 0.949874 |
| Guizhou | 2020 | 0.661805 | 0.626802 | 0.895241 | 0.185846 | 0.263099 | 0.188459 | 0.174556 | 0.271445 |
| Yunnan | 2020 | 0.379817 | 0.801517 | 0.938201 | 0.310299 | 0.515478 | 0.124909 | 0.052983 | 0.600216 |
| Shaanxi | 2020 | 0.514377 | 0.746616 | 0.506562 | 0.543607 | 0.541531 | 0.032085 | 0.599624 | 0.166813 |
| Gansu | 2020 | 0.907134 | 0.606059 | 0.631442 | 0.069739 | 0.150204 | 0.563526 | 0.062443 | 0.222385 |
| Qinghai | 2020 | 0.708468 | 0.630516 | 0.635607 | 0.328207 | 0.065677 | 0.563526 | 0.086583 | 0.052535 |
| Ningxia | 2020 | 0.148037 | 0.617745 | 0.357741 | 0.472022 | 0.065735 | 0.274209 | 0.198907 | 0.095233 |
| Xinjiang | 2020 | 0.467322 | 0.160034 | 0.488023 | 0.356497 | 0.173799 | 0.051168 | 0.034867 | 0.291927 |
| Tianjin | 2021 | 0.062691 | 0.756263 | 0.052133 | 0.892374 | 0.713536 | 0.759451 | 0.765006 | 0.235621 |
| Beijing | 2021 | 0.051278 | 0.998103 | 0.587019 | 0.988218 | 0.695624 | 0.871705 | 0.99395 | 0.318801 |
| Hebei | 2021 | 0.917425 | 0.823462 | 0.857601 | 0.547475 | 0.866662 | 0.124909 | 0.505798 | 0.23357 |
| Shanxi | 2021 | 0.911598 | 0.848463 | 0.419407 | 0.562296 | 0.415909 | 0.188459 | 0.172857 | 0.104777 |
| Inner Mongolia | 2021 | 0.831435 | 0.912135 | 0.151942 | 0.72771 | 0.35494 | 0.051168 | 0.072897 | 0.033414 |
| Liaoning | 2021 | 0.375082 | 0.419413 | 0.135024 | 0.678849 | 0.665753 | 0.032085 | 0.510642 | 0.113574 |
| Jilin | 2021 | 0.672808 | 0.98017 | 0.259672 | 0.525335 | 0.3523 | 0.188459 | 0.447384 | 0.055978 |
| Heilongjiang | 2021 | 0.322729 | 0.292462 | 0.284053 | 0.426903 | 0.528155 | 0.124909 | 0.194844 | 0.031864 |
| Shanghai | 2021 | 0.051225 | 0.941011 | 0.248416 | 0.989679 | 0.750812 | 0.274209 | 0.950507 | 0.969734 |
| Jiangsu | 2021 | 0.248377 | 0.973821 | 0.383607 | 0.909859 | 0.946078 | 0.840324 | 0.880822 | 0.827954 |
| Zhejiang | 2021 | 0.093136 | 0.953506 | 0.415695 | 0.941764 | 0.86863 | 0.59465 | 0.87591 | 0.8654 |
| Anhui | 2021 | 0.33756 | 0.823781 | 0.856994 | 0.631655 | 0.730681 | 0.124909 | 0.640817 | 0.661169 |
| Fujian | 2021 | 0.298704 | 0.782556 | 0.943131 | 0.846847 | 0.497887 | 0.682752 | 0.612136 | 0.582502 |
| Jiangxi | 2021 | 0.800186 | 0.316097 | 0.822618 | 0.614598 | 0.523504 | 0.032085 | 0.538543 | 0.34141 |
| Shandong | 2021 | 0.639862 | 0.947994 | 0.780153 | 0.733275 | 0.964409 | 0.82243 | 0.690787 | 0.889374 |
| Henan | 2021 | 0.827279 | 0.76512 | 0.955706 | 0.524439 | 0.949661 | 0.380675 | 0.503363 | 0.835677 |
| Hubei | 2021 | 0.781086 | 0.877799 | 0.635399 | 0.678864 | 0.782446 | 0.274209 | 0.636594 | 0.719273 |
| Hunan | 2021 | 0.964734 | 0.852864 | 0.629367 | 0.64574 | 0.828832 | 0.032085 | 0.581594 | 0.765645 |
| Guangdong | 2021 | 0.096514 | 0.595287 | 0.936008 | 0.859635 | 0.988827 | 0.080659 | 0.948042 | 0.845231 |
| Guangxi | 2021 | 0.759119 | 0.897842 | 0.965704 | 0.451254 | 0.602655 | 0.89767 | 0.331653 | 0.619492 |
| Hainan | 2021 | 0.283449 | 0.949809 | 0.894288 | 0.604858 | 0.060418 | 0.59465 | 0.500786 | 0.119278 |
| Chongqing | 2021 | 0.849136 | 0.94514 | 0.613199 | 0.722152 | 0.504292 | 0.051168 | 0.600032 | 0.737474 |
| Sichuan | 2021 | 0.974552 | 0.938645 | 0.774562 | 0.585239 | 0.919007 | 0.380675 | 0.620028 | 0.924254 |
| Guizhou | 2021 | 0.676232 | 0.695202 | 0.867724 | 0.321305 | 0.291831 | 0.188459 | 0.396441 | 0.383975 |
| Yunnan | 2021 | 0.395283 | 0.858828 | 0.981298 | 0.501861 | 0.547072 | 0.032085 | 0.150386 | 0.586166 |
| Shaanxi | 2021 | 0.529713 | 0.781637 | 0.777894 | 0.616111 | 0.584384 | 0.080659 | 0.6329 | 0.107602 |
| Gansu | 2021 | 0.909675 | 0.702928 | 0.864546 | 0.140933 | 0.195288 | 0.080659 | 0.187845 | 0.123047 |
| Qinghai | 2021 | 0.742524 | 0.600492 | 0.827146 | 0.503108 | 0.062338 | 0.654461 | 0.131428 | 0.043288 |
| Ningxia | 2021 | 0.138374 | 0.671729 | 0.299052 | 0.561915 | 0.089532 | 0.188459 | 0.281149 | 0.13048 |
| Xinjiang | 2021 | 0.622463 | 0.142005 | 0.423965 | 0.530166 | 0.27101 | 0.051168 | 0.066423 | 0.154382 |

Note：To meet the computational requirements of the dynamic QCA algorithm, calibrated values of 0 were replaced with a negligible constant (0.0001) to enable the successful execution of the procedure.
